# SR‐B1‐Mediated Transplacental Transfer of Hydrophobic Toxicants Disrupts Fetal Development During Barriergenesis

**DOI:** 10.1002/advs.202515742

**Published:** 2026-02-25

**Authors:** Yixuan Huang, Hailin Shang, Ling Jiao, Ce Chen, Zehua Liu, Yi Yang, Qiannan Zhang, Song Tang, Xudong Jia, Hui Yang, Yi Wan

**Affiliations:** ^1^ Laboratory For Earth Surface Processes College of Urban and Environmental Sciences Peking University Beijing China; ^2^ NHC Key Laboratory of Food Safety Risk Assessment China National Center For Food Safety Risk Assessment Beijing China; ^3^ China CDC Key Laboratory of Environment and Population Health Chinese Center for Disease Control and Prevention Beijing China

**Keywords:** gestational exposure, mass spectrometry imaging, metabolic disruption, placental development, spatial lipidomics

## Abstract

Gestation represents a critical developmental window of fetal vulnerability to xenobiotic‐induced disruptions of biological homeostasis. While xenobiotic transfer is primarily studied through maternal‐fetal comparisons at delivery, its spatiotemporal distribution across gestation stages, particularly the transport mechanisms in early pregnancy, remains poorly understood. In this study, we reveal spatiotemporal heterogeneity of hydrophobic toxicants (including persistent organic pollutants such as medium‐chain chlorinated paraffins [MCCPs] and hexabromocyclododecane) and endogenous metabolites in the developing placenta and fetus by mass spectrometry imaging (MSI). The stage of transition to hemotrophic nutrition is found as a critical window with fetal‐to‐maternal distribution ratios of xenobiotics approximately 8.4–38.2‐times higher than those estimated at birth. At this critical stage, Scavenger Receptor Class B Member 1 (SR‐B1) is identified as the key, stage‐specific, and predominate transporter mediating fetal delivery of hydrophobic toxicants and lipids in early gestation through integration of spatial metabolomics and single‐nucleus RNA sequencing of placental tissue. Finally, our findings demonstrate that early gestational exposure to MCCPs is associated with fetal neuro‐lipotoxicity. The results highlight the urgent need to reduce early‐pregnancy xenobiotic exposure for preventing toxicant‐associated fetal lipid metabolic disruption.

## Graphical abstract

This study maps the spatiotemporal distribution of hydrophobic toxicants and endogenous metabolites in the developing placenta and fetus. By integrating mass spectrometry imaging with snRNA‐seq, we identify SR‐B1 as a key transporter driving early‐gestation xenobiotic transfer and reveal a critical exposure window associated with persistent fetal metabolic disruption.

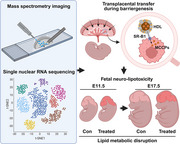

## 1 Introduction

The gestational period represents a critical developmental window during which the fetus undergoes essential metabolic processes while remaining vulnerable to disruptions in biological homeostasis [1, 2]. This concept is formalized in the Developmental Origins of Health and Disease (DOHaD) hypothesis, which posits that environmental exposures during sensitive periods of intrauterine development can led to a spectrum of chronic health disorders across multiple life stages, including infancy and adulthood [3, 4]. Epidemiological studies have raised concerns regarding the associations between prenatal environmental exposures and adverse health outcomes in offspring [5, 6, 7]. Global occurrences of hydrophobic persistent organic pollutants (POPs) at elevated concentrations in maternal, placental, and fetal blood demonstrated their substantial maternal transfer and potential developmental risks [8, 9]. Elucidating both the critical exposure windows and transport mechanisms of these hydrophobic xenobiotics is essential for understanding their developmental impacts and underlying pathogenic pathways.

The placenta serves as a critical interface between maternal tissues and the developing embryo, exhibiting remarkable cellular heterogeneity and undergoing substantial molecular and histological transformations throughout pregnancy [10, 11, 12]. Single‐cell RNA sequencing studies of murine placenta have elucidated complex cellular specification, differentiation, and maturation processes during placental development, while spatiotemporal transcriptomic atlas analyses have further revealed extensive cellular and spatial heterogeneity [13, 14, 15, 16]. In contrast, most maternal‐fetal transfer studies have primarily focused on comparative analyses between maternal and fetal samples to evaluate transplacental transfer of xenobiotics at or near delivery [17, 18, 19]. However, there remains a significant knowledge gap regarding the dynamic changes in xenobiotic and structural molecules across different gestational stages, as well as limited data on chemical accumulation within placental tissues. Notably, trophoblast cell populations progressively mature into terminally differentiated cells during pregnancy, establishing a selective permeability barrier between maternal and fetal circulation [13]. This developmental process may drive time‐dependent variations in transfer efficiency of xenobiotics, potentially influencing associated developmental risks [19]. These findings underscore the urgent need for comprehensive early‐life exposure assessments.

The placental proteins responsible for the transport of xenobiotics are of significant concerned due to their potential role in increasing the risk of adverse birth outcomes [20]. These transporters primarily belong to two major superfamilies: adenosine triphosphate‐binding cassette (ABC) transporters and solute carrier (SLC) transporters [21, 22]. Most studies focused on role of these proteins facilitating transplacental deliver of various drugs and endogenous molecular (e.g., cAMP, carnitine, steroids) [21, 23]. It is generally acknowledged that passive diffusion serves as a significant mechanism for the transplacental transfer of hydrophobic xenobiotics and lipids, particularly for compounds smaller than 600 Da [17, 21, 24, 25, 26, 27]. Recently, both the ABC and SLC transporters are found to be associated with transplacental transfer of some xenobiotics (such as perfluorooctane sulfonate, perfluorooctanoic acid, bisphenol A‐glucuronide, and liquid crystal monomers) [19, 23, 28, 29, 30, 31]. However, these studies have typically relied on molecular docking simulations, cell monolayer model, or term placental perfusion models to identify the major transporters of these chemicals at or near delivery, leaving a gap in understanding transport mechanisms during early pregnancy [19, 24, 28, 29, 30, 31].

Among the hydrophobic xenobiotics, medium‐chain chlorinated paraffins (MCCPs) were one of the chemicals with the notable concentrations (14.0–1,006 ng mL^−1^) detected in humans [17, 18, 32, 33]. In this study, we constructed spatiotemporal distribution profiles of MCCPs during murine embryonic development using a tetraphenylphosphonium chloride (Ph_4_PCl)‐enhanced mass spectrometry imaging (MSI) method. Spatial co‐localization analyses of hydrophobic toxicants (MCCPs and hexabromocyclododecane [HBCD] as model compounds) with endogenous metabolites in the placenta and analysis of reported single‐nucleus RNA sequencing (snRNA‐seq) data help to screen the proteins responsible for the spatiotemporal heterogeneity of these compounds in developing placenta. In vitro/in vivo experiments and molecular docking were applied to validate the identified proteins and clarify the transport mechanisms. The toxicant‐associated persistent disruption of fetal neural lipid homeostasis at critical window were finally assessed. This work established a comprehensive framework for assessing health impacts of hydrophobic toxicants on maternal‐fetal systems.

## 2 Results

### 2.1 Spatiotemporal Heterogeneity of Hydrophobic MCCPs in Developing Placenta and Fetus

**FIGURE 1:**
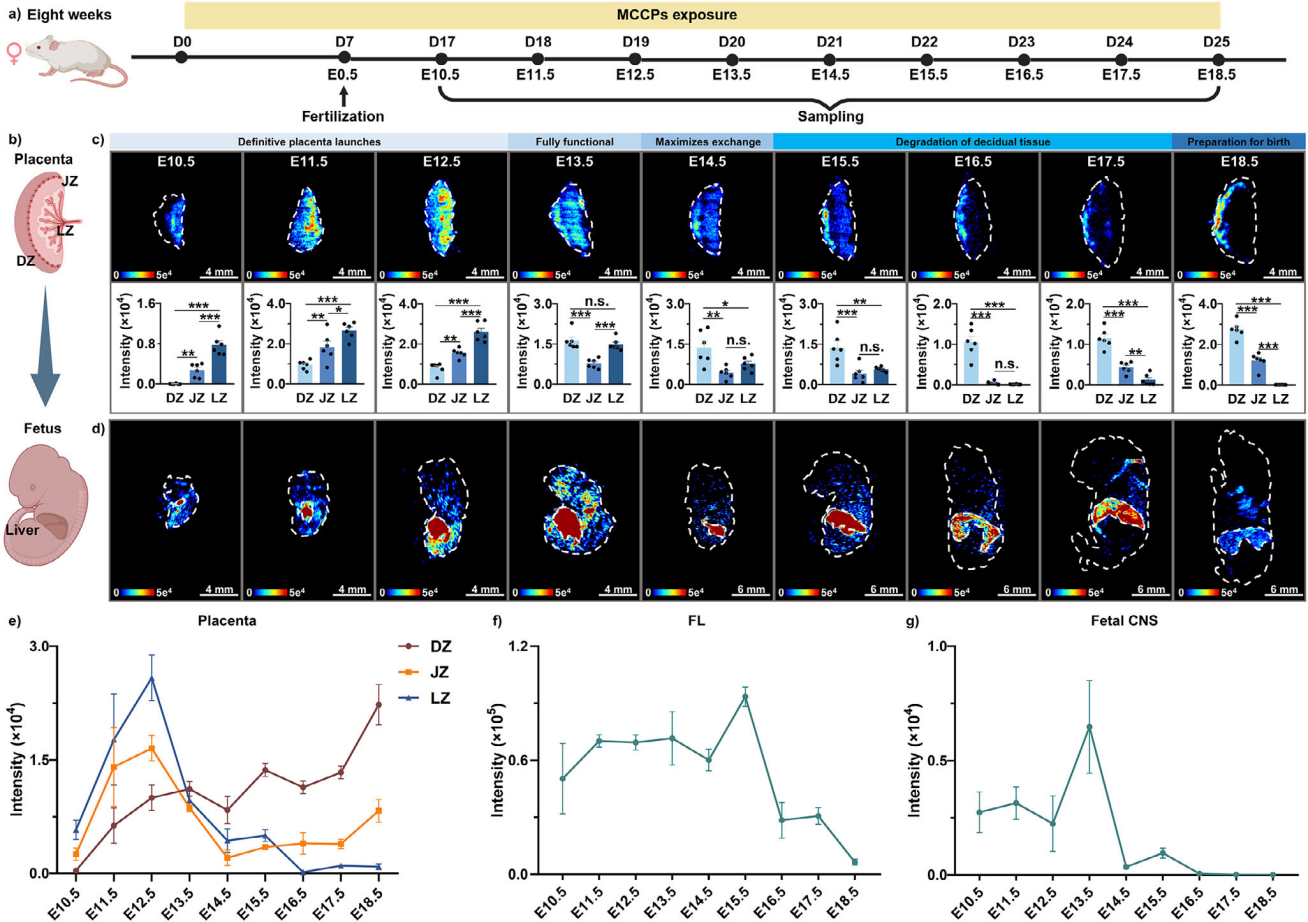
Spatiotemporal heterogeneity of hydrophobic MCCPs (representative congener: C_16_H_27_Cl_7_) in the developing placenta and fetus. (a) Schematic of the animal experimental design (Created with BioRender.com, Agreement number: NZ29540WQL). In the timeline, day zero (D0) marks the start of exposure, and day seven (D7) indicates that females were exposed for 7 days prior to mating. (b) Structure of the mouse placenta and location of the FL. (c) MS images and intensity of MCCPs in the developing placenta (*n* = 6 independent section regions were selected across three biological replicates). (d) Spatial distribution of MCCPs in the developing fetus. (e–g) Dynamic trends of MCCP intensity in distinct placental regions (e), the FL (f) and the fetal CNS (g) (three biological replicates per development timepoint). Data represent mean ± SEM. n.s., not significant; *p < 0.05, **p < 0.01, ***p < 0.001. MCCPs, medium‐chain chlorinated paraffins; DZ, decidual zone; JZ, junctional zone; LZ, labyrinth zone; FL, fetal liver; CNS, central nervous system.

We first developed an MSI method—Ph_4_PCl‐enhanced ionization coupled with air flow‐assisted ionization (AFAI)‐Orbitrap mass spectrometry—which can simultaneously visualize the spatial distributions of polyhalogenated xenobiotics and endogenous metabolites via simply adding Ph_4_PCl to the spray solvent [34]. The established calibration curves of MCCP congeners using the MSI analysis satisfied the guild line of an analytical method (*R* ^2^ > 0.99; Figure S1) [35]. The method showed high sensitivity (limit of detection: 23–840 pg mL^−1^) with no significant matrix interference in placenta and fetus sections for chlorinated paraffins (CPs) imaging (Figure S2). To investigate the transplacental transfer of hydrophobic MCCPs (24 imageable congeners as model compounds in this study; Table S1), female mice were exposed to the toxicants starting one week before pregnancy and throughout gestation (Figure 1a, mouse experiment 1). Placentas and corresponding fetuses were collected daily from E10.5 to E18.5 for Ph_4_PCl‐enhanced AFAI‐MSI analysis to visualize the transplacental transfer of MCCPs, and their representative H&E staining images are shown in Figure S3. Surprisingly, all MCCP congeners exhibited significant spatial heterogeneity within the developing placenta, with distinct distribution patterns across three zones: the decidual zone (DZ), the junctional zone (JZ), and the labyrinth zone (LZ) (Figure 1b,c, Figures S4a, and S5a). During E10.5‐E12.5, intensities of MCCPs remained highest in the LZ, followed by the JZ and DZ (Figure 1c, and Figures S4a and S5a). However, a notable shift occurred after E13.5: the DZ accumulated the highest intensities of MCCPs, exceeding those in both the LZ and JZ (Figure 1c, Figures S4a and S5a). This pattern persisted from E14.5 to E16.5, where the DZ maintained significantly elevated MCCP intensities while intensities in the LZ and JZ were comparable (Figure 1c, Figures S4a and S5a). By late gestation (E17.5–E18.5), the distribution pattern of MCCPs had completely reversed compared to the early stages (E10.5–E12.5), with the DZ showing the highest MCCP intensities, followed by the JZ and LZ (Figure 1c, Figures S4a and S5a). These results revealed a dynamic and temporally regulated spatial distribution pattern of MCCPs within the placenta, suggesting stage‐specific roles of these zones in transport and accumulation of all MCCP congeners.

The spatial distribution of MCCPs in fetus tissues was also imaged at each developmental stage corresponding to the placenta (E10.5‐E18.5). The highest intensities of MCCPs were consistently observed in the fetal liver (FL) during all stages examined (Figure 1d, Figures S4b and S5b). Relatively high intensities of MCCPs were detected in the central nervous system (CNS) tissues during early development (E10.5–E13.5). In contrast, lower intensities of MCCPs were distributed to other tissues during later stages (E14.5–E18.5).

The dynamic trends of MCCPs in the DZ, JZ, LZ, and FL from E10.5 to E18.5 were further analyzed. The intensities of MCCPs in the DZ exhibited a consistent increasing trend throughout gestation (Figure 1e, Figures S4c and S5c). In contrast, intensities of MCCP in the JZ and LZ peaked at E12.5 followed by significant declines (Figure 1e, Figures S4c and S5c). Notably, MCCP intensities in the LZ decreased sharply from E12.5 to E18.5, reducing to approximately 0.03‐fold of peak intensity, suggesting maximal exposure occurs prior to complete formation of the placental barrier genesis. Correspondingly, intensities of MCCP in the FL reached their maximum at E15.5, three days lag after the peak in the placental LZ, and decreased to 0.07‐fold of peak intensity by E18.5 (Figure 1f, Figures S4d and S5d). Additionally, the intensity of MCCPs in the fetal mouse CNS peaks at E13.5, followed by a rapid decline (Figure 1g, Figures S4e and S5e). To our knowledge, this is the first study to visualize the transplacental transfer of xenobiotics within the developing placenta and fetus, highlighting that the period from E10.5 to E12.5 represents a critical window of unexpected high exposure of MCCPs during pregnancy.

### 2.2 Spatial Co‐Localization of Hydrophobic Toxicants and Endogenous Lipids in the Placenta

We further imaged endogenous metabolites in the placenta, leveraging the capability of Ph_4_PCl‐enhanced AFAI‐MSI to simultaneously image polyhalogenated xenobiotics and endogenous metabolites. Significantly, compared with analyses without Ph_4_PCl addition, Ph_4_PCl‐enhanced ionization improved the analytical sensitivities of monoacylglycerol (MG), diacylglycerol (DG), and ceramide (Cer) by approximately 1.73–2.50‐fold, 6.62–8.83‐fold, and 2.41–11.76‐fold in the mouse brain slices, respectively (Figures S6 and S7). The endogenous lipids (e.g., phosphatidylethanolamine [PE], DG, and fatty acid [FA]) in the placenta also exhibit similar spatiotemporal heterogeneity compared to MCCPs during E10.5–E14.5, with significantly high intensities observed in the LZ (Figure S8). During E16.5–E18.5, spatial heterogeneity of these lipids disappeared (Figure S8).

**FIGURE 2:**
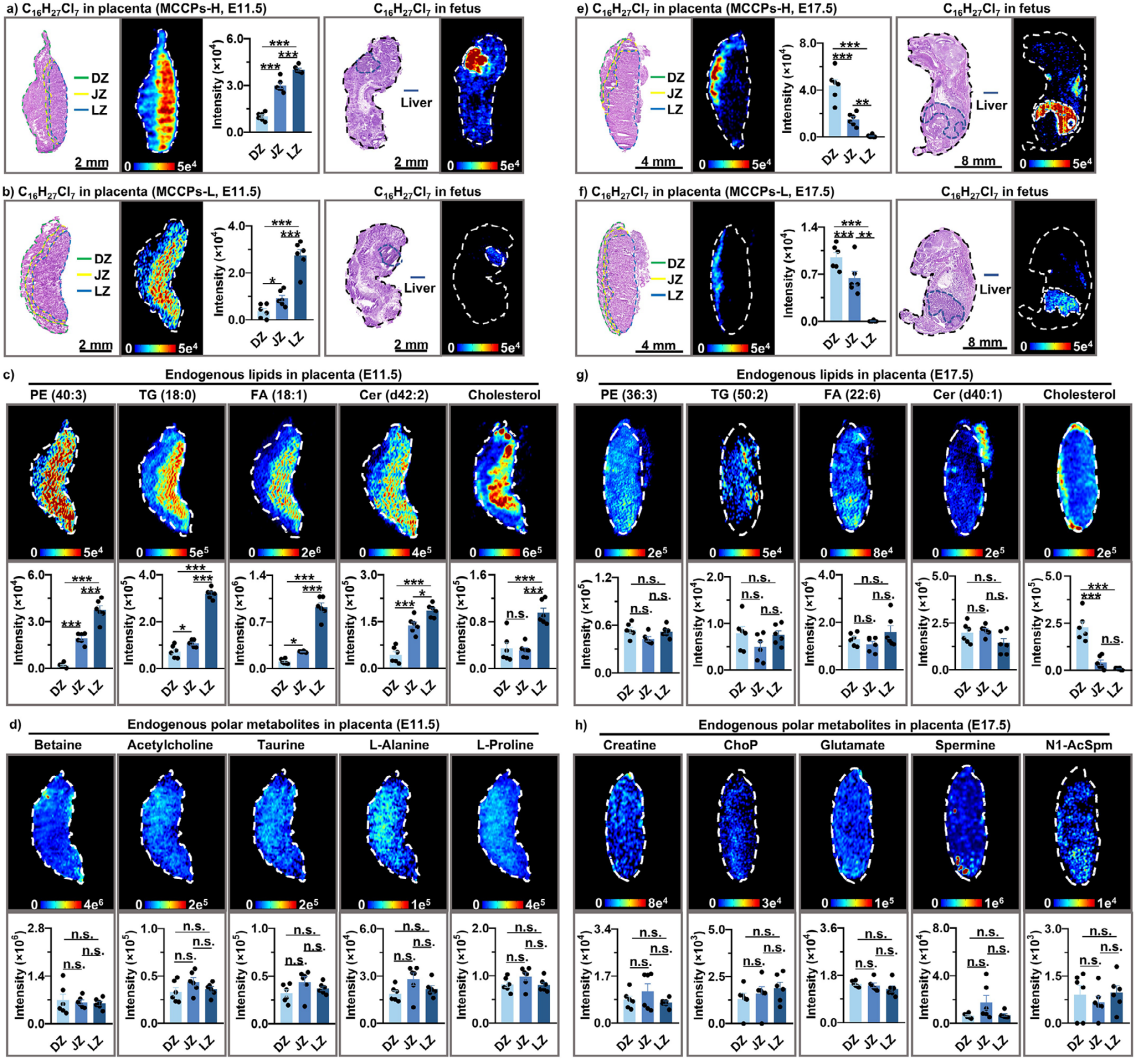
Spatial distributions of hydrophobic MCCPs (representative congener: C_16_H_27_Cl_7_) and endogenous lipids in the placenta at E11.5 and E17.5. (a,b) H&E‐stained sections, MS images, and intensity of MCCPs in the placenta and fetus at E11.5 in the MCCPs‐H group (a), MCCPs‐L group (b). (c,d) MS images and intensity of endogenous lipids and polar metabolites in the placenta at E11.5 in the MCCPs‐L group. (e,f) H&E‐stained sections, MS images and intensity of MCCPs in the placenta and fetus at E17.5 in the MCCPs‐H group (e), MCCPs‐L group (f). (g,h) MS images and intensity of endogenous lipids and polar metabolites in the placenta at E17.5 in the MCCPs‐L group. Given the variation in metabolite abundance across developmental stages, distinct metabolites were selected for analysis at E11.5 and E17.5 [40]. *n* = 6 independent section regions were selected across four biological replicates. Data represent mean ± SEM. n.s., not significant; *p < 0.05, **p < 0.01, ***p < 0.001. MCCPs, medium‐chain chlorinated paraffins; DZ, decidual zone; JZ, junctional zone; LZ, labyrinth zone; PE, phosphatidylethanolamine; TG, triglyceride; FA, fatty acid; Cer, ceramide; ChoP, phosphorylcholine; N1‐AcSpm, N1‐acetylspermine.

We focused on two critical timepoints, E11.5 and E17.5, as the distribution of MCCPs in the placenta exhibited completely reversed patterns at these stages. Mice were exposed to MCCPs at low (MCCPs‐L) and high doses (MCCPs‐H), and placental and fetal tissues were collected at E11.5 and E17.5 for imaging analysis (mouse experiment 2). We analyzed the concentrations of MCCPs and HBCD in FLs, and the concentrations of MCCPs and HBCD were 13.52 ± 6.06 and 0.66 ± 0.30 µg g^−1^, respectively, in the low dose exposure groups. Using previously reported liver‐to‐blood partition coefficients of MCCPs and HBCD [36, 37], the blood concentrations of MCCPs and HBCD in fetus were estimated to be 65.32 and 68.87 ng g^−1^, respectively. These levels are comparable to those reported in human blood (MCCPs: 3.76–3200 ng g^−1^; HBCD: 8–856 ng g^−1^), suggesting its environmental relevance [18, 38, 39]. At E11.5, significantly higher intensities of MCCPs were consistently observed in the placental LZ, followed by the JZ and DZ, in both dose groups (Figure 2a,b). Our spatial metabolomics analysis revealed that, at E11.5, hydrophobic lipids, including triglycerides (TG), Cer, FA, PE, and cholesterol, also exhibited the highest intensities in the LZ, mirroring the distribution pattern of MCCPs at this stage (Figure 2c). But hydrophilic metabolites such as betaine, taurine, and alanine exhibited a uniform distribution across the placenta, showing no spatial heterogeneity (Figure 2d). In contrast, this distribution pattern of MCCPs was completely reversed at E17.5 (Figure 2e,f). At this stage, neither lipids nor polar metabolites exhibited significant spatial heterogeneity in the placenta, with the exception of cholesterol (Figure 2g,h). The intensities of cholesterol were significantly higher in the DZ than in the LZ, a distribution pattern consistent with that of MCCPs. Importantly, the spatial distributions of visualized endogenous metabolites in placenta were unaffected by MCCP exposures, showing similar patterns in both exposed and control groups (Figures S9 and S10). These findings indicated that endogenous lipids and hydrophobic xenobiotics were co‐transported across the LZ to the fetus at E11.5, however, once the placental barrier is established at E17.5, the transport of MCCPs was significantly hindered, possibly sharing common pathways with cholesterol at this later stage.

This observation is further supported by the transplacental distribution patterns of another hydrophobic xenobiotic, HBCD (a brominated flame retardant) (Figure S11). At E11.5, HBCD accumulated in the placental LZ at higher levels compared to the JZ and DZ across two dose groups (Figure S11a,b), but this pattern was completely reversed at E17.5‐a phenomenon similarly observed for MCCPs (Figure S11c,d). Notably, the distribution patterns of HBCD resembled those of lipids but differed from polar metabolites in placentas at E11.5, while at E17.5, they were only similar to cholesterol (Figure S11e–h). The similar phenomenon of hydrophobic model compounds (MCCPs and HBCD) suggested that hydrophobic xenobiotics may pose significant fetal exposure risks during early gestation, as they can be co‐transported with endogenous lipids to the developing fetus during placental barriergenesis.

### 2.3 Identification of Proteins Responsible for Placental Transport of Hydrophobic Xenobiotics and Lipids

**FIGURE 3:**
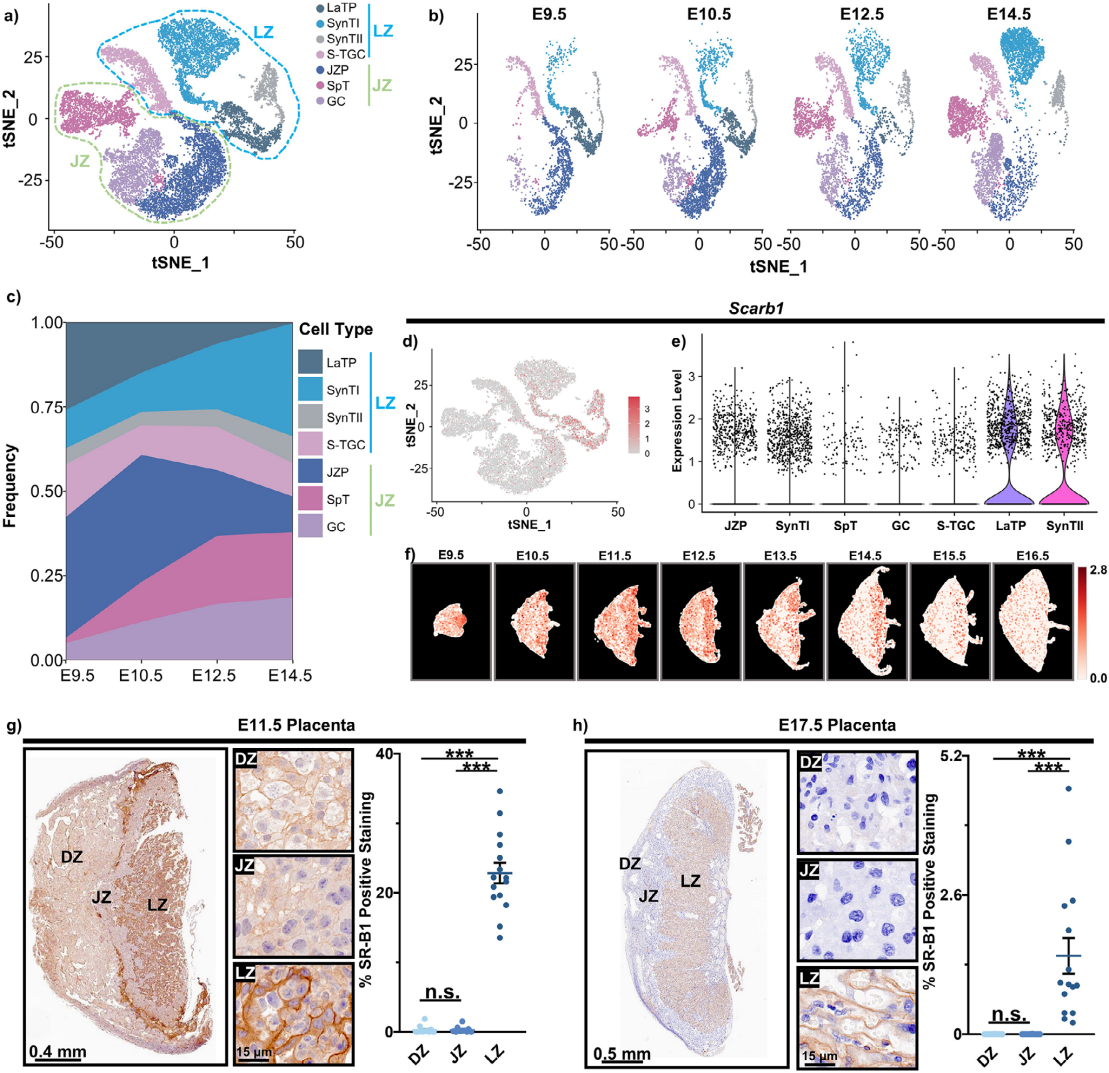
Identification of placental transport proteins responsible for placental transport of hydrophobic toxicants and lipids. (a) t‐SNE projection visualizing transcriptome similarity of the 16 386 trophoblast nuclei analyzed (Dataset: GSE152248). (b) t‐SNE plot stratified by gestational age, showing trophoblast nuclei captured at each timepoint. (c) Proportion of trophoblast clusters at each developmental stage. (d,e) *Scarb1* expression (E9.5–E14.5) visualized on t‐SNE projections (d) and summarized as a violin plot (e). (f) Spatiotemporal expression profile of *Scarb1* in the developing placenta (E9.5–E16.5) generated using the STAMP (Spatiotemporally‐resolved Transcriptome Atlas of Mouse Placenta) tool. (g‐h) IHC staining of SR‐B1 in the placenta at E11.5 (g) and E17.5 (h) (*n* = 15 regions analyzed; 5 biological replicates, 3 regions randomly selected per replicate). Data represent mean ± SEM. n.s., not significant; *p < 0.05, **p < 0.01, ***p < 0.001. t‐SNE, t‐distributed stochastic neighbor embedding; DZ, decidual zone; JZ, junctional zone; LZ, labyrinth zone; LaTP, labyrinth trophoblast progenitors; SynTΙ, syncytiotrophoblasts Ι; SynTΙΙ, syncytiotrophoblasts ΙΙ; S‐TGC, sinusoidal trophoblast giant cells; JZP, junctional zone precursors; SpT, spongiotrophoblasts; GC, glycogen cells; *Scarb1*, Scavenger Receptor Class B Member 1; IHC, Immunohistochemistry.

To identify the protein responsible for the spatial heterogeneity of hydrophobic xenobiotics in the placenta, we re‐analyzed a recently published snRNA‐seq dataset of mouse placental LZ development [13]. Given that trophoblasts are placenta‐specific cells responsible for most placental functions, we focused on 16,386 trophoblast nuclei from the original dataset via t‐distributed stochastic neighbor embedding (t‐SNE) analysis. The t‐SNE analysis revealed four distinct trophoblast subpopulations in the LZ: labyrinth trophoblast progenitors (LaTP), syncytiotrophoblasts (SynTI and SynTII), and sinusoidal trophoblast giant cells (S‐TGC); and three subpopulations in the JZ: junctional zone precursors (JZP), glycogen cells (GC), and spongiotrophoblasts (SpT) (Figure 3a). The conglomerate data was further separated by gestational timepoints to investigate developmental dynamics. LaTP populations exhibited a rapid developmental decline, becoming nearly undetectable by E14.5 (Figure 3b). Conversely, terminally differentiated cell populations (SynTI, SynTII, and S‐TGC) showed significant expansion (Figure 3b). Quantification of each cell population across gestational timepoints revealed an inverse relationship between progenitor (LaTP) and differentiated cell populations (SynTI, SynTII, and S‐TGC) during placental development (E9.5–E14.5) (Figure 3c). While LaTP progressively diminished, their differentiated progeny (SynTI, SynTII, and S‐TGC) undergo substantial increases (Figure 3c). Notably, the fetal‐maternal blood barrier is composed of three layers of trophoblast cells: SynTII, SynTI, and S‐TGC [13]. Thus, candidate proteins mediating the differential accumulation of hydrophobic toxicants (e.g., MCCPs or HBCD) and endogenous lipids in the LZ—high at E11.5 but low after E14.5—should exhibit high expression in LaTP cells but low expression in SynTI, SynTII, or S‐TGC populations. Using these criteria, we initially screened 50 lipid‐related candidates and narrowed the selection to 7 proteins based on t‐SNE projection similarities (Figure 3d, Figure S12). Among these, only Scavenger Receptor Class B Member 1 (SR‐B1) has a known role in lipid transport (particularly cholesterol) via interacting with high‐density lipoprotein (HDL) [41]. Its expression across trophoblast subpopulations in t‐SNE space aligned with the expected differential trends, supporting its potential role in early placental transport of hydrophobic compounds (Figure 3d,e).

To validate the spatial expression pattern of *Scarb1* in the placenta, we first utilized the Spatiotemporally‐resolved Transcriptome Atlas of Mouse Placenta (STAMP) tool to visualize the spatiotemporal distribution of *Scarb1* in the developing placenta (E9.5‐E16.5) (Figure 3f), which is similar to that of the endogenous lipids (Figure S8) [16]. We further conducted immunohistochemistry (IHC) staining on paraffin‐embedded placental sections at E11.5 and E17.5. SR‐B1 exhibited predominant membrane‐associated localization (Figure 3g,h). At E11.5, expression of SR‐B1 was significantly higher in the LZ compared to both the DZ and JZ (Figure 3g), consistent with the elevated levels of hydrophobic xenobiotics (e.g., MCCPs or HBCD) and endogenous lipids observed in the LZ at this stage. At E17.5, SR‐B1 showed minimal expression in the DZ and JZ (Figure 3h), potentially restricting the translocation of hydrophobic xenobiotics and cholesterol from the DZ to the LZ. The relatively high expression of SR‐B1 detected in the LZ at E17.5 (Figure 3h) was primarily attributed to its expression in the SynTII subpopulation (Figure 3e). Additionally, we assessed the expressions of proteins potentially mediating maternal transfer of xenobiotics and lipids, such as transthyretin (*TTR*) and microsomal triglyceride transfer protein (*MTTP*) [42, 43]. t‐SNE projections revealed negligible expression of *Ttr* and *Mttp* across all trophoblast clusters (Figure S13).

### 2.4 SR‐B1‐Mediated Transplacental Transfer of Hydrophobic Xenobiotics In Vitro

**FIGURE 4:**
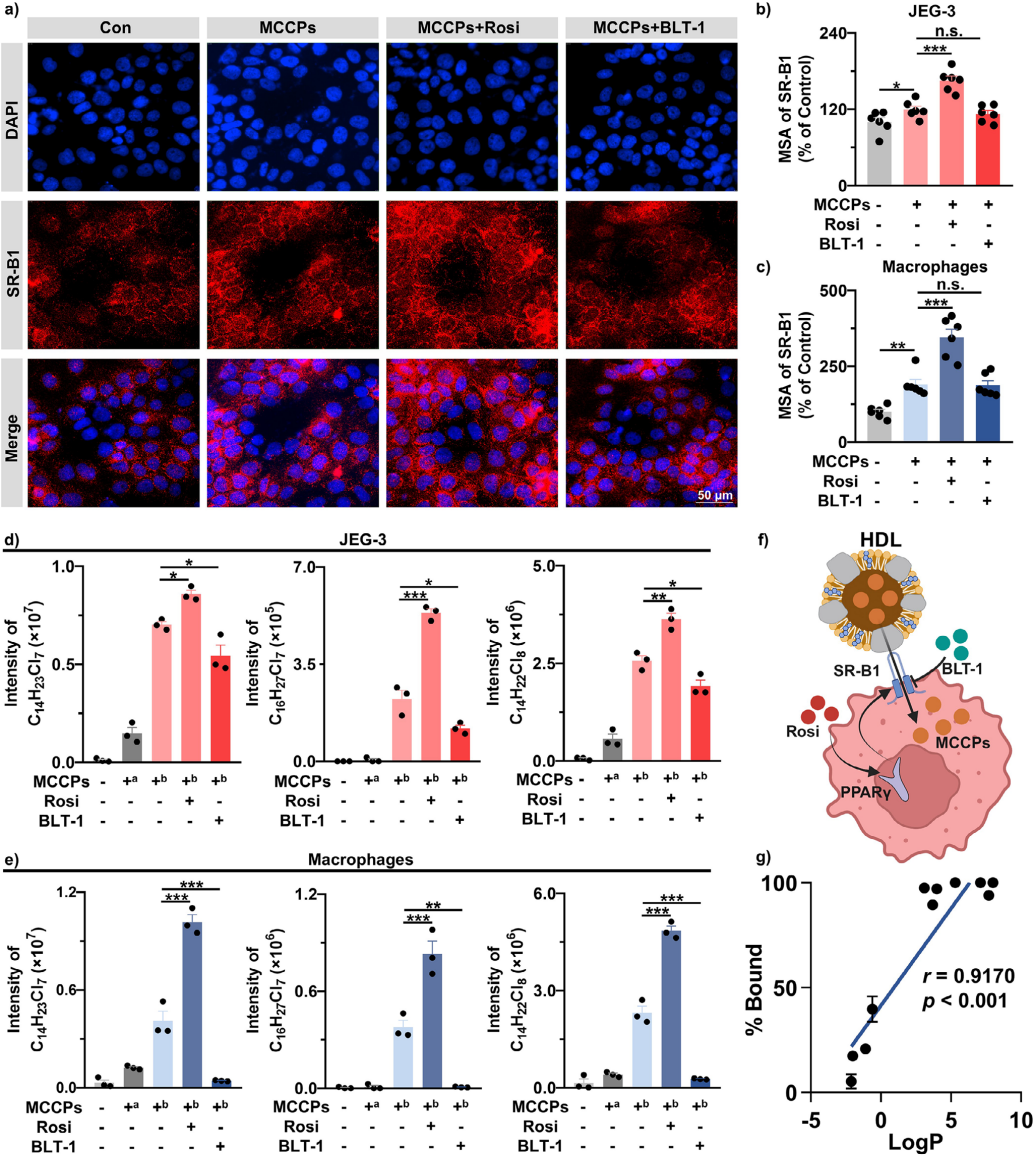
SR‐B1‐mediated transplacental transfer of hydrophobic MCCPs in vitro. (a) Representative images of SR‐B1 expression in JEG‐3 cells treated with vehicle control, MCCPs, MCCPs+Rosi, or MCCPs+BLT‐1. (b,c) Expression levels of SR‐B1 protein in JEG‐3 cells (b) and macrophages (c) treated with vehicle control, MCCPs, MCCPs+Rosi, or MCCPs+BLT‐1 (*n* = 6 biological replicates per group). No significant cytotoxicity was observed (Figure S16). (d,e) Intracellular MCCPs intensity in JEG‐3 cells (d) and macrophages (e) treated with vehicle control, MCCPs, MCCPs+Rosi, or MCCPs+BLT‐1 (*n* = 3 biological replicates per group). (a) exposure for 10 min; (b) exposure for 72 h. (f) Schematic diagram of the proposed transport mechanism (Created with BioRender.com, Agreement number: SI29540VVW). (g) Correlation between binding affinity with HDL and chemical lipophilicity (*n* = 3 replicates per chemical). Pearson correlation coefficient was calculated. Data represent mean ± SEM. n.s., not significant; *p < 0.05, **p < 0.01, ***p < 0.001. MCCPs, medium‐chain chlorinated paraffins; Rosi, rosiglitazone; BLT‐1, block lipid transport‐1; SR‐B1, Scavenger Receptor Class B Member 1; MSA, mean stained area; HDL, high‐density lipoprotein; PPARγ, Peroxisome Proliferator‐Activated Receptor γ.

In vitro experiment was conducted to investigate the role of SR‐B1 in mediating transplacental transfer of hydrophobic MCCPs. We assessed expressions of SR‐B1 in JEG‐3 cells (a human placental choriocarcinoma cell line) and macrophages (which typically exhibit SR‐B1 expression) using high‐content imaging. No significant signals of SR‐B1 were observed in the negative controls (Figure S14). Exposure of MCCPs upregulated SR‐B1 expression to 1.20‐fold or 1.90‐fold of control levels in JEG‐3 and macrophages, respectively (Figure 4a–c, Figures S15 and S16). Rosiglitazone (Rosi) is a known PPARγ agonist, and its activation upregulates SR‐B1 expression [44, 45]. Co‐exposure with Rosi enhanced SR‐B1 expressions beyond MCCP exposure alone (Figure 4a–c, Figures S15 and S16), suggesting that both MCCPs and Rosi can induce expression of SR‐B1, with Rosi exerting more pronounced effects. In contrast, co‐exposure of MCCPs and block lipid transport‐1 (BLT‐1) (SR‐B1 transport inhibitor) resulted in SR‐B1 expression levels comparable to MCCP exposure alone (Figure 4a–c, Figures S15 and S16), consistent with BLT‐1's known mechanisms of binding SR‐B1 to block its transport function without affecting expression [46]. The results were further validated by western blot assays (Figures S17 and S18).

We next evaluated SR‐B1's role in mediating MCCP transport in JEG‐3 and macrophages. A 10‐minute MCCP exposure control confirmed that the detected MCCP levels represented true cellular uptake rather than surface adsorption. Significantly higher intracellular concentrations of MCCPs were observed in JEG‐3 (Figure 4d, Figure S19a) and macrophages (Figure 4e, Figure S19b) co‐exposed to MCCPs and Rosi compared with the MCCPs‐exposure groups. Conversely, co‐treatment with MCCPs and BLT‐1 resulted in significant reductions in intracellular MCCP concentrations in both JEG‐3 (Figure 4d, Figure S19a) and macrophages (Figure 4e, Figure S19b) compared with the MCCPs‐exposure groups. Notably, the magnitude of MCCP concentration variations between treatment groups was greater in macrophages than JEG‐3 (Figure 4d,e, Figure S19), consistent with the higher basal expression of SR‐B1 (a member of CD36 superfamily of scavenger receptors) in macrophages [47, 48, 49]. These results established SR‐B1 as a key transporter facilitating cellular MCCP uptake.

Molecular docking simulations were performed to elucidate the binding mechanisms of SR‐B1 with hydrophobic xenobiotics (MCCPs and HBCD). Although BLT‐1 has been reported to bind directly to SR‐B1 at Cys384, our results revealed distinct interaction sites for MCCPs and HBCD [50]. Chlorine atoms in MCCPs formed hydrogen bonds with the –NH group of Arg98 and the –NH_2_ group of Gln418 in SR‐B1 (Figure S20a), and bromine atoms in HBCD formed hydrogen bonds with the OE1 atom of Glu115 in SR‐B1 (Figure S20b). The results implied that MCCPs and HBCD did not directly bind to SR‐B1. Given the well‐established role of SR‐B1 in HDL‐cholesterol transport and the similar spatiotemporal distribution patterns shared by MCCPs, HBCD, and cholesterol in the placenta (Figure 2, Figures S9–S11), these hydrophobic xenobiotics were possibly transported through the SR‐B1‐HDL mediated pathway (Figure 4f). We further performed rapid equilibrium dialysis (RED) to evaluate binding affinity between HDL and chemicals with varying lipophilicity. Eleven chemicals, including pipemidic acid (PPA), amoxicillin (AMX), ampicillin (AMP), sulfanilamide (SA), roxithromycin (ROM), mono‐(2‐ethylhexyl) phthalate (MEHP), dieldrin, mirex, *syn*‐dechlorane plus (*syn*‐DP), MCCPs, and HBCD were selected. The HDL‐bound chemical fraction were measured to be 94% and 100% for MCCPs and HBCD, respectively. Similarly, the HDL‐bound fractions of ROM, MEHP, dieldrin, mirex, and *syn*‐DP were as high as 90%‐100%, confirming that these hydrophobic compounds could bind to HDL with high‐affinity. In comparison, relatively low HDL‐bound fractions (5%‐40%) were obtained for PPA, AMX, AMP, and SA, and a significant positive correlation was observed between the bound fraction and chemical lipophilicity (*r* = 0.9170, *p* < 0.001) (Figure 4g).

### 2.5 SR‐B1‐Mediated Transplacental Transfer of Hydrophobic Xenobiotics and Lipids In Vivo

**FIGURE 5:**
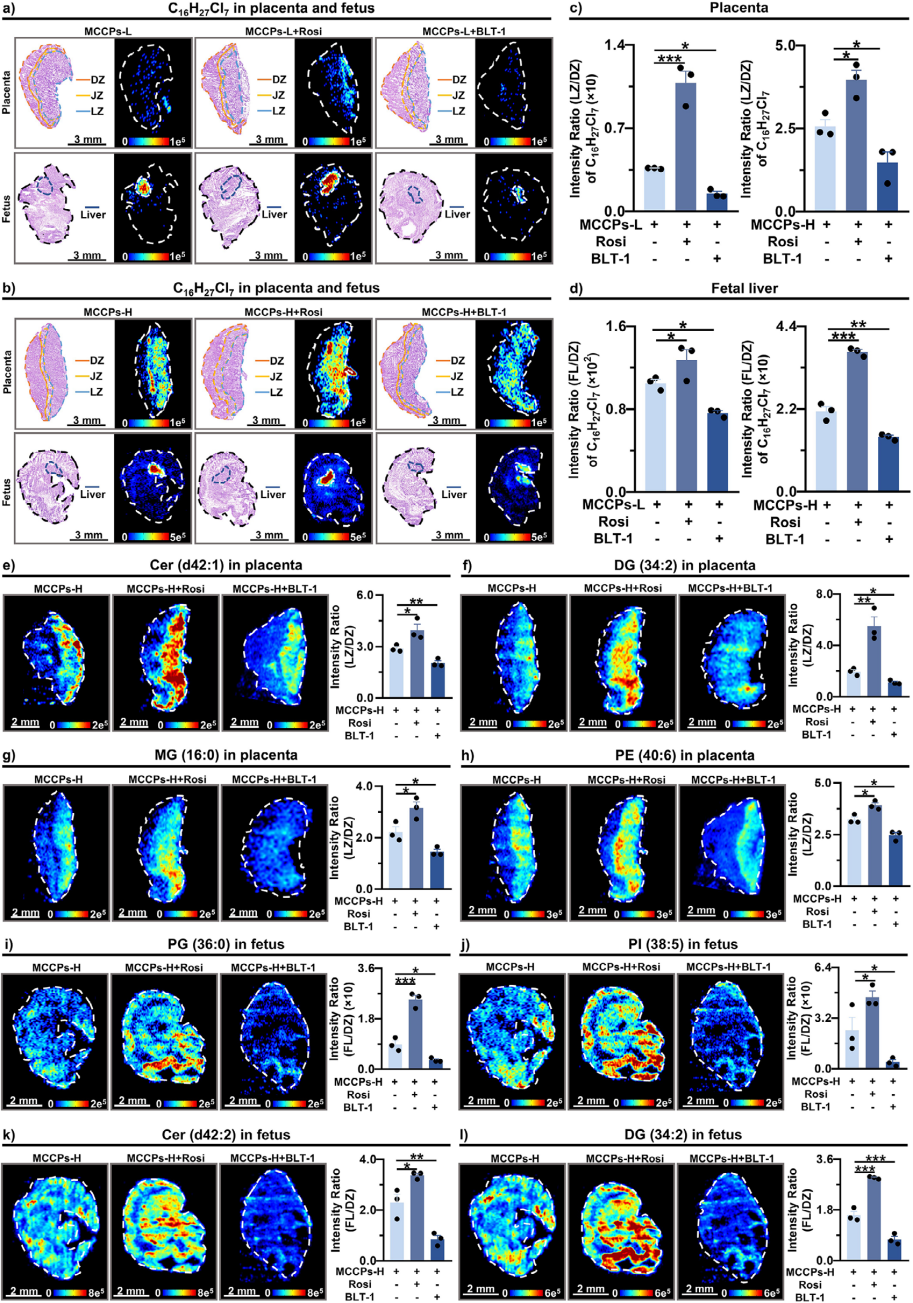
SR‐B1‐mediated transplacental transfer of hydrophobic MCCPs and lipids in vivo. (a,b) H&E‐stained sections and MS images of MCCPs in the placenta and fetus at E11.5 in groups treated with low‐dose MCCPs alone or in combination of Rosi/BLT‐1 (a) and high‐dose MCCPs alone or in combination of Rosi/BLT‐1 (b). (c) Intensity ratio (LZ/DZ) of MCCPs in the placenta at E11.5 across treatment groups (*n* = 3 biological replicates per group). (d) Intensity ratio (FL/DZ) of MCCPs in FL at E11.5 across treatment groups (*n* = 3 biological replicates per group). (e–h) MS images and intensity ratio (LZ/DZ) of lipids in the placenta at E11.5 in high‐dose treatment groups (*n* = 3 biological replicates per group). (i–l) MS images and intensity ratio (FL/DZ) of lipids in fetus at E11.5 in high‐dose treatment groups (*n* = 3 biological replicates per group). Data represent mean ± SEM. *p < 0.05, **p < 0.01, ***p < 0.001. MCCPs, medium‐chain chlorinated paraffins; Rosi, rosiglitazone; BLT‐1, block lipid transport‐1; DZ, decidual zone; JZ, junctional zone; LZ, labyrinth zone; FL, fetal liver; Cer, ceramide; DG, diacylglycerol; MG, monoglyceride; PE, phosphatidylethanolamine; PG, phosphatidylglycerol; PI, phosphatidylinositol.

To validate the role of SR‐B1 in mediating the maternal transfer of MCCPs during the early stage, an in vivo experiment was conducted by gavaging pregnant mice with MCCPs, along with either Rosi or BLT‐1 (mouse experiment 3). The distribution patterns of MCCPs in the DZ, JZ, LZ, and FL were compared across groups at E11.5. The intensity ratios of LZ/DZ of MCCPs in the placenta were calculated to correct the potential variations in maternal MCCP concentrations. Co‐exposure of MCCPs and Rosi increased the ratios, whereas co‐exposure to MCCPs and BLT‐1 decreased them, compared to MCCPs exposure group (Figure 5a–c, Figures S21a–c and S22a–c). Consistently, the intensity ratios of FL relative to DZ (FL/DZ) of MCCPs showed similar changes. Co‐treatment with MCCPs and Rosi significantly increased the intensity ratios of MCCPs compared with the MCCPs groups, whereas co‐treatment with MCCPs and BLT‐1 reduced the intensity ratios (Figure 5d, Figures S21d and S22d).

Given SR‐B1's role in lipid transport during barriergenesis, spatial lipidomics was performed on both the placenta and fetus alongside SR‐B1 activation/inhibition. Co‐treatment with MCCPs and Rosi significantly increased the intensity ratios of LZ/DZ of placental lipids compared to MCCPs exposure groups, such as Cer (d42:1), DG (34:2), MG (16:0), and PE (40:6) (Figure 5e–h). In contrast, co‐treatment with MCCPs and BLT‐1 significantly reduced the intensity ratios of LZ/DZ of these lipids compared to MCCPs exposure groups (Figure 5e–h). Consistently, intensity ratios of FL/DZ of lipids in the fetus exhibited similar changes upon SR‐B1 activation or inhibition (Figure 5i–l, Figure S23). Co‐treatment with MCCPs and Rosi significantly increased the intensity ratios of multiple lipids, while co‐treatment with MCCPs and BLT‐1 decreased them. These coordinated in vivo changes in lipids and MCCPs confirmed that SR‐B1 activation/inhibition regulated the transport of both hydrophobic xenobiotics and lipids from the placenta to the fetus during barriergenesis.

### 2.6 Early‐Stage Exposure of Hydrophobic Toxicants was Associated With Persistent Disruption of Fetal Neural Lipid Homeostasis

**FIGURE 6:**
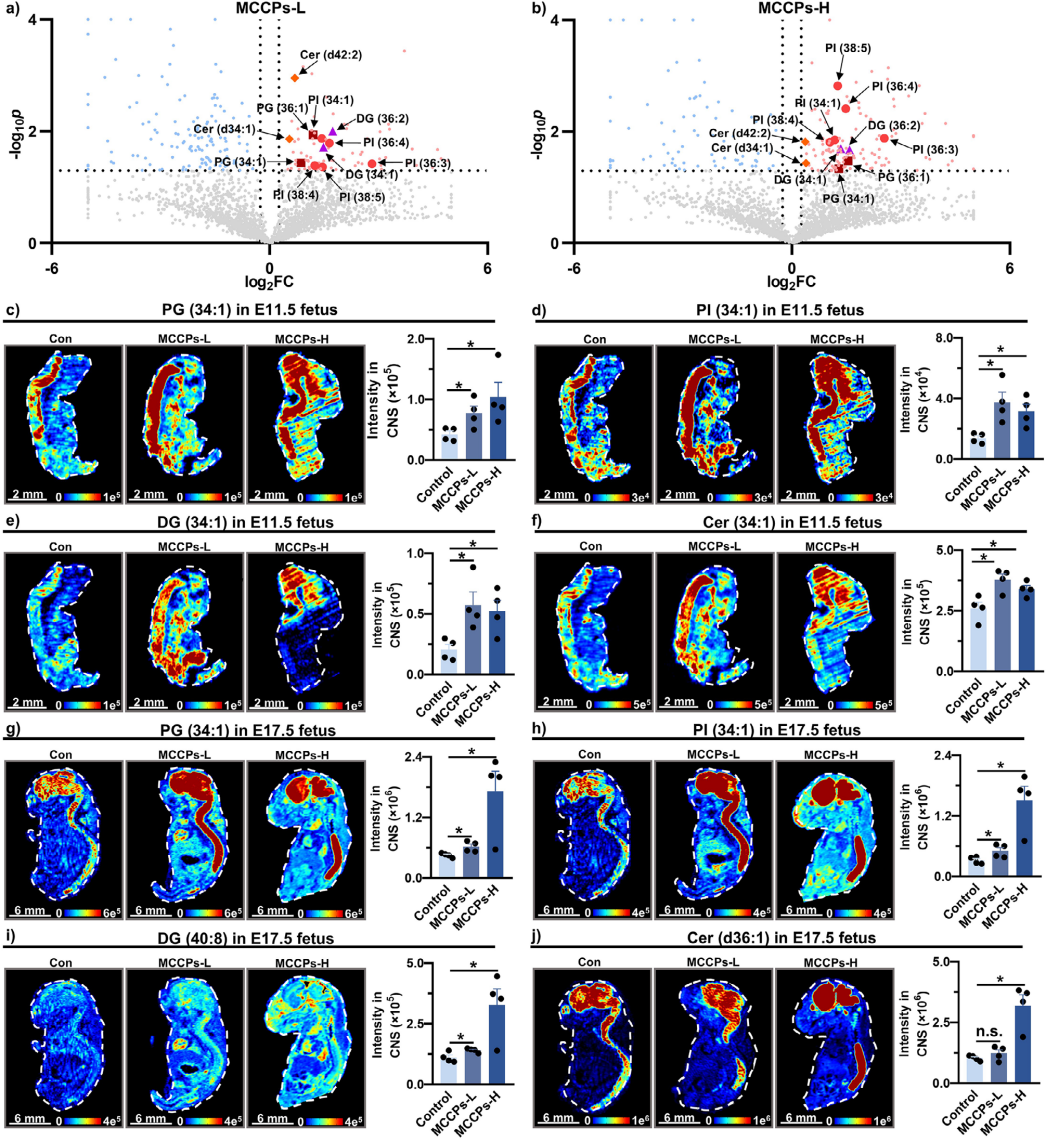
Early‐stage exposure of hydrophobic toxicants was associated with persistent disruption of fetal neural lipid homeostasis. (a,b) Volcano plots of differentially endogenous lipids analyzed by MSI: (a) MCCPs‐L versus control, (b) MCCPs‐H versus control. (c–f) MS images and intensity of toxic lipids in CNS of fetus at E11.5 across treatment groups (*n* = 4 biological replicates per group). (g–j) MS images and intensity of toxic lipids in CNS of fetus at E17.5 across treatment groups (*n* = 4 biological replicates per group). Data represent mean ± SEM. n.s., not significant; *p < 0.05, **p < 0.01, ***p < 0.001. MCCPs, medium‐chain chlorinated paraffins; PG, phosphatidylglycerol; PI, phosphatidylinositol; DG, diacylglycerol; Cer, ceramide; CNS, central nervous system.

Next, this study conducted spatial metabolomic analysis on MCCPs‐exposed fetus to evaluate the metabolic disruption effects following early‐stage exposure (mouse experiment 2). Volcano plot analysis indicated that multiple phosphatidylglycerol (PG), phosphatidylinositol (PI), Cer, and DG species exhibited significantly elevated concentrations in CNS (including brain and spinal cord) in both low‐ and high‐dose MCCPs‐exposed groups compared to controls at E11.5 (Figure 6a,b) [51]. Imaging analysis showed obvious changes in these lipids in CNS. The intensities of these lipids in CNS were significantly increased to 1.46–7.05‐fold and 1.29–5.82‐fold in the low‐dose and high‐dose groups, respectively, relative to controls (Figure 6c–f, Figure S24). The increased levels of so‐called toxic lipids (sphingolipid, glyceride, and glycerophospholipids) in CNS in E11.5 was more obvious at E17.5. Low‐dose MCCPs exposure led to a 1.38–3.39‐fold increase in the abundance of PG, PI, and DG, and high‐dose exposure significantly increased the intensities of PG, PI, Cer, and DG (2.67–7.66‐fold) (Figure 6g–j, Figure S25). The rapid brain expansion and the formation of distinct compartments occurring at E11.5 indicate that this stage represents a critical neuro‐developmental window [52]. We further validated the lipotoxic effects of MCCPs on CNS in cell models. Exposure of MCCPs induced a significant increase in lipid abundance in BV2 cells without significant cytotoxicity (Figure S26). Thus, maternal exposure to MCCPs at an early stage (E11.5) was associated with lipotoxic effects in CNS, with disruption of fetal neural lipid homeostasis persisted through E17.5.

We also evaluated the spatial metabolic alterations induced by HBCD in fetal mice. Spatially resolved metabolomics revealed that exposure to both low and high doses of HBCD led to a significant decrease in the intensities of uridine diphosphate glucose, formamidopyrimidine nucleoside triphosphate, and D‐threito in the liver of fetal mouse, with these effects persisting from E11.5 to E17.5 (Figure S27). This indicated that dysregulation of hepatic glucose‐related metabolism in fetuses was associated with maternal exposure of HBCD. Collectively, these results demonstrated that early‐stage exposure to hydrophobic xenobiotics was associated with developmental metabolic disruption in fetal mice, although the effects vary across different toxicants.

## 3 Discussion

Our study was the first, to our knowledge, to visualize toxicants during gestation and reveal their spatiotemporal heterogeneity, along with endogenous metabolites, in the developing placenta. In addition, we successfully identified SR‐B1 as a transporter for hydrophobic toxicants and lipids across the placenta to the fetus before the formation of transplacental barrier. Finally, our findings demonstrated that the exposure burden during the critical window (E10‐E12: stage of transition to hemotrophic nutrition) is significantly higher than previously estimated, which was associated with persistent developmental metabolic disruption.

Biomonitoring studies evaluating prenatal toxicant exposures typically analyzed maternal biospecimens (e.g., blood, urine, hair) and fetal samples (e.g., cord blood, amniotic fluid, meconium), comparing their concentrations to assess transplacental transfer and maternal exposure [1, 21, 53]. These studies only captured placental transfer at or near delivery, lacking dynamic changes in the transplacental transportation of environmental toxicants during pregnancy. CPs, among the most highly polluted chemicals in humans, have demonstrated maternal transfer in pregnant women, with cord‐maternal ratios of 0.36–1.25 for SCCPs and 0.11–2.20 for MCCPs [17, 18, 32]. In this study, the in situ imaging method successfully delineated the spatiotemporal dynamics of MCCPs in placental and fetal tissues throughout gestation. The results showed that fetal‐to‐maternal distribution ratios of MCCPs at E11.5 (1.29–20.5) were approximately 8.4–38.2 times higher than those at E18.5 (0.07–1.28) (Figure 1). This remarkably elevated transplacental transport efficiency could be attributed to the accumulation of these compounds (including CPs and HBCD) into HDL‐associated lipid core and their subsequent transport to the fetus via SR‐B1 during transplacental barriergenesis (Figure 3). Notably, POPs are groups of hydrophobic toxicants and have attracted global attention [54]. While reported fetal‐to‐maternal ratios for most POPs ranged from 0.3 to 6.3, studies relying exclusively on post‐gestational investigations likely substantially underestimated both fetal exposure burdens and potential toxicological consequences of these chemicals [7, 53, 55].

The placenta serves as a crucial protective organ that sustains fetal development by regulating nutrient and metabolite exchange between maternal and fetal circulations, while simultaneously functioning as a selective barrier against xenobiotic exposure [30, 56]. Recently, several studies focused on cellular heterogeneity, gene expression patterns, and morphological variations along the maternal‐fetal axis during placentation [11, 13, 14, 15, 16]. To examine changes of metabolites within the placental tissue microenvironment, the entire placental tissue was generally homogenized for measurement, obscuring crucial information on spatial localization [57, 58]. Ph_4_PCl‐enhanced AFAI‐MSI developed by this study showed high sensitivity for visualizing small molecules, enabling simultaneous mapping of endogenous metabolites and hydrophobic toxicants in the developing placenta [34]. Strikingly, these molecules exhibited pronounced spatiotemporal heterogeneity during placental development, especially, lipids showed different distribution patterns between the JZ, DZ, and LZ at E11.5 versus E17.5 (Figure 2). These findings aligned with the established transition from histiotrophic to hemotrophic nutrition during early gestation [11]. Specifically, the period between E10 and E12 represents the onset of hemotrophic nutrition—a labyrinth‐dependent, vascular‐mediated process where nutrients (including lipids) are transferred via countercurrent exchange from maternal sinusoids to fetal capillaries [11]. This study found that this hemotrophic phase not only facilitated efficient maternal‐fetal nutrient transfer but also enabled unexpectedly high transport efficiency of toxicants alongside endogenous lipids prior to full maturation of the placental barrier. This was validated by the maximal exposure in fetus observed occurs prior to complete formation of placental barriergenesis (Figure 1). Consequently, the stage of transition to hemotrophic nutrition (E10–E12) was demonstrated as a critical developmental window for fetal exposure to toxicants.

As a dynamically remodeling organ, the placenta undergoes structural maturation and evolving membrane during development, which can produce time‐ and zone‐dependent differences in passive permeability [11]. Spatial distribution patterns of endogenous lipids revealed microenvironmental shifts that would modulate partitioning and consequent diffusion kinetics (Figure S8). In fact, passive diffusion has been considered as a primary mechanism for the transplacental transfer of hydrophobic compounds [21]. But this mechanism alone does not fully explain the observed spatiotemporal heterogeneity of both hydrophobic xenobiotics and lipids in placenta in this study (Figures 1 and 2). We systematically screened all proteins involved in the transport of lipid and hydrophobic xenobiotics during the stage of transition to hemotrophic nutrition (E10–E12), and identified SR‐B1 as a key mediator of transplacental transfer of hydrophobic compounds. SR‐B1 binds HDL with high affinity, facilitating the transport of liposoluble molecules, most notably the cellular uptake of cholesteryl esters from cholesterol‐rich HDL particles [46, 59, 60, 61, 62]. The similar transfer behaviors observed for MCCPs and HBCD, coupled with their similar placental distribution patterns to cholesterol (Figure 2, Figures S9–S11), strongly indicate that these environmental contaminants accumulate within the lipid core of HDL particles. They are subsequently co‐transported into trophoblast cells via the SR‐B1‐mediated pathway (Figure 4f). The significant positive correlation between chemical lipophilicity and HDL binding affinity (Figure 4g) indicates that diverse hydrophobic chemicals can bind to HDL with high affinity and undergo SR‐B1‐mediated transplacental transport. Therefore, a chemical's specific HDL affinity would determine its fetal exposure burden in early gestation. SR‐B1 is highly expressed in the liver and steroidogenic tissues, and it is also a lipoprotein receptor expressed in placenta as an HDL receptor [47, 62]. It plays a pivotal role in maternal‐fetal cholesterol exchange within the placenta [41, 63, 64]. SR‐B1‐deficient mouse embryos exhibit a high incidence of exencephaly and intrauterine growth restriction [65]. Our results revealed that SR‐B1‐mediated lipid supply during the stage of transition to hemotrophic nutrition may inadvertently facilitate the transfer of hydrophobic toxicants to the fetus. While the spatial heterogeneity does not exclude a substantial contribution from passive diffusion, the SR‐B1‐mediated transport mechanism plays a key, stage‐specific role, predominating during early gestation and therefore causing a remarkable increase in transplacental transport efficiency for hydrophobic toxicants. In later developmental stages, the fetus acquired autonomous lipid synthesis capacity, thus, the placental barrier formed alongside diminished SR‐B1 expression in DZ and JZ (Figure 3), hindering the transfer of hydrophobic toxicants and lipids (Figure 1, Figure S8) [66]. This downregulation of placental SR‐B1 represented a critical event in placental barrier establishment.

High exposure to toxicants (e.g., MCCPs) during the stage of transition to hemotrophic nutrition (E10‐E12) could induce significant developmental metabolic disruption. Using MSI technology, we found that MCCP exposure prior to placental barrier formation led to excessive deposition of toxic lipids (e.g., ceramide, diacylglycerol) in the fetal CNS, with effects persisting throughout gestation [67, 68]. The excessive accumulation of these lipids triggers cellular distress and dysfunction; hence they are termed “toxic lipids” [67]. For example, the observed elevated ceramides may potentially induce endoplasmic reticulum stress and lipotoxicity in the developing fetal brain [69]. This lipotoxicity was further validated in BV2 microglial cells (Figure S26), consistent with previous reports of short‐chain chlorinated paraffin (SCCP)‐induced lipid upregulation in the same cell line [70]. Such excessive intracellular lipid accumulation could directly induce mitochondrial dysfunction and oxidative stress [71]. Supporting these findings, existing neurotoxicity studies on CPs have shown that chemical exposure in zebrafish embryos impaired locomotor capacity in larvae [72]. Our study provided the first direct evidence that early gestational exposure to hydrophobic toxicants was associated with persistent disruption of fetal neural lipid homeostasis, highlighting the critical importance of mitigating toxicant exposure during pregnancy to prevent metabolic dysregulation and disease predisposition in offspring.

This research is subject to several limitations. First, the integration of our semi‐quantitative MSI data with a public snRNA‐seq dataset should be considered hypothesis‐generating, as trophoblast transcriptional states in the placenta could be influenced by potential differences in strain, diet, and husbandry. Secondly, the “lipid‐related” candidate screening may overlook the potential roles of other placental transporters (e.g., ABC/SLC families). Thirdly, it should be cautious when extrapolating the findings to humans as this study primarily relies on a mouse model. Fourthly, the study lacks definitive downstream functional or structural endpoints, and future functional studies are needed to establish neurodevelopmental toxicity.

## 4 Conclusion

Taken together, this study presented the first spatiotemporal atlas tracking hydrophobic toxicants and endogenous metabolites across gestational development in both placenta and fetus. By integrating spatial co‐localization analysis with snRNA‐seq of placental tissue, we identified SR‐B1 as the key transporter mediating the transfer of hydrophobic toxicants and lipids to the fetus prior to placental barrier formation. Our findings revealed that the stage of transition to hemotrophic nutrition constituted a critical developmental window for both lipid uptake and inadvertent exposure to hydrophobic toxicants, and was associated with persistent developmental metabolic disruption.

## 5 Experimental Section

### 5.1 Chemicals and Reagents

Medium‐chain chlorinated paraffins (MCCPs, 52% Cl), short‐chain chlorinated paraffins (SCCPs, 55.5% Cl), dieldrin, and mirex were purchased from Dr. Ehrenstorfer (Augsburg, Germany). Hexabromocyclododecane (HBCD) was obtained from Macklin (Shanghai, China). *Syn*‐dechlorane plus (*Syn*‐DP) was purchased from Accustandard (New Haven, USA). ^13^C_10_‐*anti*‐DP was obtained from Cambridge Isotope Laboratories (Shanghai, China). Rosiglitazone (Rosi), block lipid transport‐1 (BLT‐1), and phorbol 12‐myristate 13‐acetate (PMA) were provided by MCE (Shanghai, China). Carboxymethylcellulose (CMC) was supplied by Coolaber (Beijing, China). Tetraphenylphosphonium chloride (Ph_4_PCl), mono‐(2‐ethylhexyl) phthalate (MEHP), and dimethylsulfoxide (DMSO) were purchased from Sigma‐Aldrich (St. Louis, USA). LC‐MS grade methanol (MeOH) and acetonitrile (ACN) were obtained from Merck (Darmstadt, Germany). Methyl tert‐butyl ether (MTBE), formic acid (FA), BCA Protein Assay Kit, and ECL chemiluminescence kit were provided by ThermoFisher (Waltham, USA). Ultrapure water was prepared by a Milli‐Q Synthesis water purification system (Millipore, Bedford, MA, USA). Pipemidic acid (PPA), amoxicillin (AMX), ampicillin (AMP), sulfanilamide (SA), and roxithromycin (ROM) were purchased from Alta Scientific (Tianjin, China). Paraformaldehyde (4%) and Hoechst 33342 were supplied by Solarbio (Beijing, China). Roswell Park Memorial Institute (RPMI) 1640 medium, Dulbecco's modified Eagle's medium (DMEM), fetal bovine serum (FBS), penicillin/streptomycin (P/S), and phosphate‐buffered saline (PBS) were purchased from Gibco (New York, USA). Anti‐scavenging receptor SR‐BI antibody was obtained from Abcam (Shanghai, China. RRID: AB_3696478). Anti‐rabbit lgG (H+L) F(ab') Fragment (Alexa Fluor 555 Conjugate), anti–β‐actin antibody, HRP‐linked anti‐rabbit IgG secondary antibody, Cell Lysis Buffer, and 10X TBST were provided by Cell Signaling Technology (Boston, USA). Protease inhibitor was purchased from Roche (Basel, Swiss). HCS LipidTOX Green Phospholipidosis Detection Reagent (1000X), Bis‐Tris gels, and iBlot 2 Transfer Stacks were supplied by Invitrogen (Waltham, USA). Triton and QuickBlock were purchased from Beyotime (Shanghai, China). Human high‐density lipoprotein (HDL) was obtained from Yuanye Bio‐Technology (Shanghai, China). For cell lines, Human choriocarcinoma JEG‐3 cells (purchased in 2024. RRID: CVCL_0363) and murine microglial BV2 cells (purchased in 2024. RRID: CVCL_0182) were purchased from National Collection of Authenticated Cell Cultures (Shanghai, China). Human monocytic THP‐1 cells (purchased in 2018. RRID: CVCL_0006) were obtained from American Type Culture Collection (Manassas, USA). All cell lines used in the experiments were found to be contamination free.

### 5.2 Animal Experiments

CD‐1 mice (eight weeks old) were purchased from the Beijing Vital River Laboratory Animal Technology Company and acclimatized to the housing environment for two weeks before exposure. Mice were housed in standard cages under controlled temperature (22 ± 2°C) and relative humidity (40%–60%), with a 12 h light:dark cycle. Food and drinking water were provided ad libitum. Exposed chemicals were dissolved in edible corn oil and administered to female mice via gavage. All animal studies were approved by the Institutional Animal Care and Use Committee of Peking University (Approval No. Urban‐WanY‐1).

#### 5.2.1 Experiment 1: Spatiotemporal Distribution Patterns of MCCPs and Lipids in Placenta and Fetus

MCCPs were administered to female mice at dose of 500 mg kg^−1^ d^−1^ for 7 days prior to pregnancy. After the exposure period, they were mated with unexposed male mice at a 1:1 ratio. Successful mating was confirmed by the presence of a vaginal plug the following morning, designated as embryonic day 0.5 (E0.5). The pregnant mice were then transferred to fresh cages and continuously exposed to MCCPs. At E10.5, E11.5, E12.5, E13.5, E14.5, E15.5, E16.5, E17.5, and E18.5, the mice were euthanized, and their placentas and fetuses were immediately dissected from the uterus on ice. All samples were snap‐frozen in liquid nitrogen and stored at −80°C. Three placentas and three fetuses were selected at each development timepoint for subsequence analysis.

#### 5.2.2 Experiment 2: Spatial Co‐Localization Analysis of Xenobiotics and Endogenous Metabolites

Female mice were randomly divided into five groups, including control group (corn oil only), MCCPs‐L group (50 mg kg^−1^ d^−1^), MCCPs‐H group (500 mg kg^−1^ d^−1^), HBCD‐L group (10 mg kg^−1^ d^−1^), and HBCD‐H group (100 mg kg^−1^ d^−1^). Prior to pregnancy, mice were exposed to MCCPs or HBCD for 28 days. Pregnant mice continued to receive treatment until E11.5 or E17.5, after which they were euthanized. Placentas and fetuses were dissected on ice. From the control group, five E11.5 placentas and five E17.5 placentas were fixed in 4% paraformaldehyde immediately after dissection for immunohistochemical staining. The remaining samples were snap‐frozen in liquid nitrogen to prevent metabolite alterations and store at −80°C until further analysis. For each treatment group, four placentas and four fetuses were collected at each time point (E11.5 and E17.5) for MSI analysis.

#### 5.2.3 Experiment 3: Role of SR‐B1 on Maternal Transfer of Hydrophobic Toxicants In Vivo

Female mice were divided into seven experimental groups: control group (corn oil only), MCCPs‐L group (50 mg kg^−1^ d^−1^ MCCPs), MCCPs‐L+Rosi (50 mg kg^−1^ d^−1^ MCCPs + 10 mg kg^−1^ d^−1^ Rosi), MCCPs‐L+BLT‐1 (50 mg kg^−1^ d^−1^ MCCPs+1 mg kg^−1^ d^−1^ BLT‐1), MCCPs‐H group (500 mg kg^−1^ d^−1^ MCCPs), MCCPs‐H+Rosi (500 mg kg^−1^ d^−1^ MCCPs+10 mg kg^−1^ d^−1^ Rosi), and MCCPs‐H+BLT‐1 (500 mg kg^−1^ d^−1^ MCCPs+1 mg kg^−1^ d^−1^ BLT‐1). The dosages of Rosi and BLT‐1 were based on previous studies [41, 73, 74]. All groups received a 7‐day pre‐pregnant exposure followed by an 8‐day gestational exposure period to MCCPs at their designated concentrations. From E8.5 to E11.5, mice in the respective groups were administered MCCPs alone or in combination with Rosi or BLT‐1, respectively. Pregnant mice were euthanized at E11.5, and placentas and fetuses were collected. All samples were immediately snap‐frozen in liquid nitrogen and stored at −80°C for subsequent analysis. Three placentas and three fetus samples from each group were selected for imaging analysis.

### 5.3 Preparation of Frozen Sections of Placenta and Fetus

Placenta and fetus samples were embedded with 3% CMC in molds, and stored at −80°C for about 2 h. The embedded samples were equilibrated at −20°C for 30 min in a cryostat chamber (Leica CM1950, Nussloch, Germany. RRID: SCR_018061) prior to sectioning. Samples were cut into serial 40 and 14 µm sections. All sections were thaw‐mounted onto glass slides and stored at −80°C until analysis. The slides with thickness of 40 µm were desiccated at room temperature for about 15 min before MSI scanning. Adjacent sections with thickness of 14 µm were stained using standard hematoxylin‒eosin (H&E) protocols.

### 5.4 Immunohistochemical Analysis

Placentas tissues collected at E11.5 and E17.5 were dissected and fixed in 4% paraformaldehyde for 24 h. Paraffin sectioning and subsequent immunohistochemical staining were performed according to standard histological procedures. Rabbit anti‐ SR‐B1 antibody (1:1600; from abcam, Shanghai, China; Catalog number: ab217318) was used. Stained sections were digitally scanned and analyzed using a high‐throughput whole‐slide imaging system (OLYMPUS, Tokyo, Japan. RRID: SCR_017564). The results were further analyzed using ImageJ software (RRID: SCR_003070).

### 5.5 AFAI‐MSI Analysis

The MSI analysis was performed following established protocols from our previous work [34]. An Air Flow Assisted Ionization (AFAI) ion source (Viktor, Beijing, China) equipped with a Q‐Exactive Plus Orbitrap mass spectrometer (Thermo Scientific, CA, USA. RRID: SCR_020556) operating in both negative and positive mode was used for MSI analysis. Scanning velocity settings were optimized according to development stages. For placentas and fetuses collected prior to E14.5, the experiment was performed by scanning the sections with a constant velocity of 100 µm s^−1^ in *x*‐direction and step size of 100 µm in *y*‐direction. For fetus samples at development stages at E14.5‐E18.5, the velocity in *x*‐direction and step size in *y*‐direction were set as 200 µm s^−1^ and 200 µm, respectively. The distance between the sprayer and the ion transporting tube, as well as that between the sprayer and the slide, was uniformly set to 2 mm. The sprayer angle was adjusted to about 65°. AFAI parameters including extracting gas, spray gas, and spray voltage were set as 40 L/min, 0.6 Mpa, and ±6000 V, respectively. Additionally, the scan range was 80–1000 Da, the MS resolution was set at 70 000, the capillary temperature was 350°C, the automated gain control (AGC) target was 1 × 10^6^, and the maximum injection time (MIT) was 200 ms. Sheath gas, aux gas, and sweep gas were all set at 0 Arb. Highly stable spray solvent was delivered by a nano‐ultra performance liquid chromatography (Nano Acquity, Waters, Milford, USA), with a constant flow rate of 5 µL min^−1^. In negative ion mode, 150 µM Ph_4_PCl dissolved in MeOH was used as spray solvent. In positive ion mode, the spray solvent consisted of a MeOH/H_2_O/FA (95/5/0.1, v:v:v) mixture. To evaluate matrix interferences, 1 µL standard solution of SCCPs (55.5% Cl)/MCCPs (52% Cl) at 1 µg mL^−1^ was dropped onto blank slides and unexposed placenta or fetus sections. After drying at room temperature, MSI analysis was performed to compare intensity differences of CPs between slides.

### 5.6 MSI Data Processing

The acquired raw data files were converted to. cdf format using Xcalibur 4.2 (Thermo Scientific, CA, USA. RRID: SCR_014593), and imported into MassImager v1.0 (Chemmind Technologies Co., Ltd., Beijing, China) for spatial ion visualization. Background subtraction was performed with a mass tolerance of 0.005 Da and a subtraction ratio of 1. H&E‐stained histological images were co‐registered with MSI data in MassImager to enable precise spatial alignment. Region‐specific MS profiles were accurately selected and extracted using “arbitrary region tool”, generating 2D matrices containing *m/z* and ion intensities. The matrices were imported into Markerview 1.2.1 (AB SCIEX, USA) for peak alignment (mass tolerance: 5 ppm; minimum response threshold: 200).

After peak alignment, signals were normalized by “Total Area Sums” method to eliminate batch effects and reduce the mass intensity variant in each mass pixel [75]. To evaluate linearity of calibration curves, a series of serially diluted MCCP (52% Cl) standard solutions (0.01, 0.1, 1, 10, and 100 µg mL^−1^) were spotted onto a clean slide, with 1 µL per spot. After drying at room temperature, the slide was analyzed using the established MSI method to assess sensitivity. The LOD for each MCCP congener was defined as the lowest concentration that yielded a detectable MSI signal. It is common practice in MSI analysis to directly compare the intensities of compounds across different tissue regions [75, 76]. The fetal‐to‐maternal distribution ratio for each MCCP congener was calculated as its intensity ratio between the fetal liver (FL) and placental DZ. For this calculation, ROIs corresponding to the placental DZ and FL at each time point were precisely delineated on the MS images, and followed by data processing using the steps described above.

The above ion list was subjected to annotation by querying public databases, including the Human Metabolome Database (HMDB, https://hmdb.ca/. RRID: SCR_007712) and LIPID MAPS (https://lipidmaps.org/. RRID: SCR_003817). Annotation criteria included an exact mass tolerance of 5 ppm and high‐resolution isotopic pattern matching. Adduct types considered for negative ion mode were [M − H]^−^, [M − H − H_2_O]^−^, [M + Cl]^−^, [M + FA − H]^−^, and [M + Hac − H]^−^. For positive ion mode, the following adducts were included: [M + H]^+^, [M + H − H_2_O]^+^, [M + Na]^+^, [M + K]^+^, and [M + NH_4_]^+^. Discriminating metabolites were screened by unpaired two‐sided student's t test at confidence intervals 0.95.

### 5.7 Cell Culture

JEG‐3 cells and THP‐1 cells were cultured with RPMI 1640 supplemented with 10% FBS and 1% P/S. THP‐1 cells were seeded in 6‐well plates (1 500 000 cells per well) or black‐wall 96‐well plates (80 000 cells per well) with culture medium containing 50 ng mL^−1^ PMA. After adhesion overnight, the cells were subjected to rest in fresh culture medium for 24 h to generate M0 macrophages. BV2 cells were cultured with DMEM containing 10% FBS and 1% P/S. All cell lines were maintained in 5% CO_2_ at 37°C.

### 5.8 Cell Treatment

MCCPs, Rosi, and BLT‐1 were individually dissolved in DMSO and diluted to the indicated concentrations using RPMI 1640 medium. The final concentration of DMSO was maintained at 0.1% (v/v) across all treatment groups, including control groups. Each treatment was performed in triplicate (*n* = 3) for all cell lines. JEG‐3 cells (300 000 cells per well) and macrophages (1 500 000 cells per well) were seeded in 6‐well plates and treated with MCCPs alone or in combination with Rosi/BLT‐1 for 72 h. The concentrations of each compound for cell treatment were set as follows: MCCPs (1 µg L^−1^), Rosi (10 µM), and BLT‐1 (5 µM for JEG‐3 cells; 1 µM for macrophages). An additional short‐term exposure group (10 min) was included for excluding potential MCCP adsorption in each cell line. Before treatment, MCCPs were measured in JEG‐3 cells and macrophages, and the compounds were undetectable in the cells.

### 5.9 Analysis of Intracellular MCCPs

Cells were rinsed five times with PBS to remove extracellular MCCPs and digested from 6‐well plates. Digestion was quenched with 1 mL culture medium, and cell suspensions were transferred to glass tubes. Samples were centrifuged at 1,200 rpm for 5 min at 4°C. Supernatants were discarded, and cell pellets were resuspended in 200 µL deionized water. Protein precipitation was initiated by adding 450 µL of ice‐cold ACN, followed by vortexing and incubation at 4°C for 15 min. Samples were then centrifuged at 7500 rpm (6000 × *g*) for 15 min at 4°C. Supernatants were transferred to fresh vials, and the remaining residue was subjected to a second extraction with 200 µL of ACN and 200 µL of MTBE. Combined supernatants were dried under vacuum using a freeze dryer. The resulting residues were reconstituted in 50 µL of ACN for LC‐MS analysis.

The analysis was performed by a Thermo Ultimate 3000 UPLC coupled with Q‐Exactive Plus Orbitrap mass spectrometer. An Agilent Poroshell 120 EC×C8 column (100 × 2.1 mm, 2.7 µm) was used for separation of chemicals. Ultrapure water (A) and methanol with Ph_4_PCl (B) were set as the mobile phases, and the concentration of Ph_4_PCl was 10 µM. The flow rate was 0.2 mL min^−1^, and the gradient elution program was designed as follows: 0–1 min, 10% B; 1–1.5 min, 10%–60% B; 1.5–3.5 min, 60% B; 3.5–7 min, 60%–100% B; 7–12 min, 100% B; 12–12.5 min, 100%–30% B; 12.5–13.5 min, 30%–10% B; 13.5–15 min, 10% B. The column temperature was 40°C, the injection volume was 3 µL, and the temperature of sample compartment was set at 4°C. Orbitrap mass spectrometer was operated in negative ion mode, and full‐scan mode was selected for data acquisition. Scan range, resolution, AGC target, and MIT were set as 100–1000, 140 000, 5 × 10^6^, and 250 ms, respectively. Parameters of heated electrospray ionization (HESI) source were set as follows: sheath gas, 35 Arb; auxiliary gas, 10 Arb; sweep gas, 1 Arb; spray voltage, 2.5 kV; capillary temperature, 200°C; S‐lens radio frequency, 60. Xcalibur (v 4.2.28.14) was used for data analysis.

### 5.10 High‐Content Imaging of SR‐B1

JEG‐3 cells (10,000 cells per well) and macrophages (80 000 cells per well) were seeded in black‐wall 96‐well plates for 24 h, followed by treatment with MCCPs alone or in combination with Rosi/BLT‐1 for 48 h. Each treatment group included six biological replicates per cell line. The concentrations of chemicals were identical to those in the section of “Cell treatment”. Following treatment, cells were rinsed with PBS, fixed with 4% paraformaldehyde for 15 min, and permeabilized with 0.25% Triton X‐100 for 15 min. After blocking with blocking buffer for 15 min, the primary antibody against SR‐B1 (1:250 in blocking buffer) was applied and incubated overnight at 4°C. Samples were then incubated with a mixture of anti‐rabbit lgG (H+L) F(ab') Fragment (Alexa Fluor 555 Conjugate) (1:1000 in blocking solution, CST, 4413s) and Hoechst 33342 (1:100 in PBS, Solarbio) for 1 h at room temperature in the dark. FluoroBrite DMEM was used to reduce background fluorescence prior to image acquisition. Fluorescent cell images were captured using the ImageXpress Micro Confocal High Content Imaging System (version 6.5, Molecular Device. RRID: SCR_020294), with at least 5 sites per well analyzed. Images of Hoechst‐stained nuclei were quantified for cell cytotoxicity assessment, and images of SR‐B1 staining were analyzed for mean stained area (MSA) quantification by using CellReporterXpress Imaging and Analysis Software (version 6.5, Molecular Device. RRID: SCR_025681).

### 5.11 Western Blot Analysis

For western blotting, JEG‐3 cells and macrophages were collected and lysed using 1× Cell Lysis Buffer (CST, 9803S) supplemented with protease inhibitor (Roche, 04693159001), then centrifuged at 12 000 × *g* for 20 min at 4°C. The supernatant was harvested, and protein concentration was determined with a BCA Protein Assay Kit (Thermo Scientific, 23227) according to the manufacturer's protocol. Identical amounts of protein were separated on 4%–12% Bis‐Tris gels (Invitrogen, NP0322BOX), and transferred to nitrocellulose membranes using iBlot 2 Transfer Stacks (Invitrogen, IB23002). The blotted membranes were blocked with 5% non‐fat milk in TBST at room temperature and incubated overnight with a mixture of anti–SR‐B1 primary antibody (diluted 1:2000, Abcam, Catalog number: ab217318) and anti‐β‐actin antibody (diluted 1:5000, CST, #4967) at 4°C, then with HRP‐linked anti‐rabbit IgG secondary antibody (CST, 7074P2, diluted 1:2000) for 1 h at room temperature. Finally, bands were detected with ECL chemiluminescence kit (Thermo Scientific, 34095) and quantified by ImageJ.

### 5.12 Rapid Equilibrium Dialysis (RED) Assay

The lipoprotein binding of chemicals was assessed using a RED system (Thermo Scientific, 90112). Each measurement was performed using three RED devices. Eleven chemicals with varying lipophilicity (PPA, AMX, AMP, SA, ROM, MEHP, dieldrin, mirex, *syn*‐DP, MCCPs, and HBCD) were selected for RED assay. Human HDL (Yuanye Bio‐Technology, S26775) was diluted with PBS to 1 mg mL^−1^, then spiked with chemicals to a final concentration of 100 ppb. Spiked HDL (300 µL) was loaded into the sample chamber, and PBS (500 µL) was added to the buffer chamber. The plate was then mixed on an incubating microplate shaker at 100 rpm and 37°C for 5 h. After the dialysis process, 50  µL of dialyzed HDL solution in sample chamber was extracted with 300  µL of ACN and 50 µL of PBS, and 50  µL of dialyzed PBS in buffer chamber was extracted with 300  µL of ACN and 50 µL of diluted HDL. The extracts were vortex‐mixed for 5 min and placed on ice for 30 min, then all the samples were centrifuged at 14 000 × *g* at 4°C for 30 min, and supernatant was further analyzed using LC‐MS/MS or Orbitrap‐LC‐MS. The percentage of the compounds bound was calculated as follows:

$$\begin{array}{ccc} \%\;\mathrm{Bound} & = & 1-\left(\text{Concentration buffer chamber/}\right.\\ & & \left.\text{Concentration sample chamber}\right)\times 100\% \end{array}$$

The LogP values of each chemical were retrieved from Pubchem (RRID: SCR_004284). A %Bound value of 100% indicates that the concentration of the chemical in the buffer chamber was below the limit of detection.

### 5.13 Analysis of RED Samples

For analysis of AMX, AMP, and MEHP, chromatographic separation and determination were performed using an ACQUITY UPLC coupled to a Xevo TQ‐XS mass spectrometer (Waters, Milford, USA). Specifically, for amoxicillin and ampicillin, the ESI‐MS/MS was operated in positive mode, and a BEH C18 column (2.1×100 mm, 1.7 µm particle size, Waters) was employed for the LC separation. The column and sampler temperatures were maintained at 40°C and 4°C, respectively. The injection volume was 5 µL. 0.1% formic acid in Milli‐Q water (A) and methanol (B) were used as the mobile phases. The elution gradient was programmed as follows: 0–1.5 min, 10%–16% B; 1.5‐9 min, 16%–100% B; 9–12 min, 100% B; 12–12.1 min, 100%–10% B; 12.1–15 min, 10% B. The flow rate was 0.30 mL min^−1^. For MEHP, the ESI‐MS/MS was operated in negative mode and a Poroshell 120 HPH C18 column (2.1 × 100 mm, 1.9 µm, Agilent) was employed for the LC separation. The column and sampler temperatures were maintained at 35°C and 4°C, respectively. The injection volume was 5 µL. 0.1% acetic acid in Milli‐Q water (A) and methanol (B) were used as the mobile phases. The elution gradient was programmed as follows: 0–1 min, 10% B; 1–1.5 min, 10%–45% B; 1.5–4 min, 45% B; 4–10 min, 45%–100% B; 10–13 min, 100%–10% B. The flow rate was 0.30 mL min^−1^. The parameters of MRM transitions, cone voltages, and collision energies are listed in Table S2.

For analysis of other chemicals, Thermo Ultimate 3000 UPLC coupled with Q‐Exactive Plus Orbitrap mass spectrometer were employed for chromatographic separation and the determination. The instrumental method was identical to that described in the “Analysis of intracellular MCCPs” section.

### 5.14 Determination of MCCPs and HBCD in Tissue Samples

FLs were dissected from E17.5 fetuses in the MCCPs‐L and HBCD‐L groups (*n* = 6 per group). After weighing, each tissue was homogenized in 600 µL of ice‐cold ACN. A 200 µL aliquot of the homogenate was centrifuged at 14 000 × *g* for 20 min at 4°C. The supernatant was collected, spiked with 10 ng of ^13^C_10_‐anti‐DP as an internal standard, and then mixed with 650 µL of ice‐cold MTBE. The mixture was vortexed for 10 min. Subsequently, 160 µL of deionized water was added, followed by another 5 min of vortexing and centrifugation at 14 000 × *g* for 20 min at 4°C. The organic supernatant was carefully transferred and dried under vacuum using a freeze dryer. The residue was reconstituted in 100 µL of ACN for analysis by Orbitrap‐LC‐MS. The same instrumental method was used and described in the section “Analysis of intracellular MCCPs”. MCCPs were quantified according to a previously established procedure [32].

### 5.15 Screening of Transport Protein Mediating Hydrophobic Compounds During Barriergenesis

This study analyzed a publicly available single‐nucleus RNA sequencing (snRNA‐seq) dataset of the mouse placental labyrinth zone reported by Marsh et al. (NCBI Gene Expression Omnibus, GSE152248) [13]. T‐distributed stochastic neighbor embedding (t‐SNE) analysis was performed to visualize the trophoblast cell populations in the dataset. Proteins with significantly higher expression in labyrinth trophoblast progenitors (LaTP) and significantly lower expression in syncytiotrophoblasts Ι/ΙΙ (SynTΙ/SynTΙΙ) or sinusoidal trophoblast giant cells (S‐TGC) were screened, with criteria of *p* < 0.01 after Benjamini‐Hochberg (BH) correction and |log_2_(fold change)| > 1. Functional annotation of the screened proteins was conducted sequentially, followed by further filtration for lipid‐related proteins. Potential transporters mediating placental transfer of hydrophobic compounds were identified by similarity in t‐SNE projections. For spatial localization of the proteins in the placenta, the interactive data portal named Spatiotemporal Transcriptomic Atlas of Mouse Placenta (STAMP) was used to visualize their spatial distribution [16].

### 5.16 Molecular Docking

The crystal structure of SR‐B1 (AF‐Q61009‐F1‐v4) was modeled by AlphaFold (RRID: SCR_025454), yielding a QMEAN score of 0.67 ± 0.05 for the predicted structure. Ramachandran plot analysis showed that 96.65% of the amino acid residues resided in the most favored regions. Three‐dimensional structures of MCCPs (exemplified by C_16_H_27_Cl_7_) and HBCD were retrieved from Pubchem. Following pretreatment of proteins and ligands, structure‐based docking was performed using MOE 2018.01 software (RRID: SCR_014882).

### 5.17 Lipotoxiciy Evaluation in Nervous Cells

MCCPs were dissolved in DMSO to prepare a 100 mg L^−1^ stock solution, which was stored at −20°C. The stock solution was first diluted 1000‐fold with DMEM to yield a working solution, followed by two‐fold serial dilutions to generate MCCP concentrations ranging from 0 to 100 µg L^−1^. BV2 cells (5000 cells per well) were seeded in black‐wall 96‐well plates for 24 h, and then treated with MCCPs for 48 h. Each treatment group included three biological replicates. After treatment, cells were rinsed with PBS, fixed with 4% paraformaldehyde for 15 min, then stained with a mixture of HCS LipidTOX Green specific for phospholipids (1:1000 in PBS, Invitrogen, H34350) and Hoechst 33342 (1:100 in PBS, Solarbio) for 30 min at room temperature in the dark to label phospholipids and nuclei, respectively. FluoroBrite DMEM was applied to minimize background fluorescence prior to image acquisition. Fluorescence imaging and MSA quantification followed the same procedures described in the “High‐content imaging of SR‐B1” section.

### 5.18 Statistical Analysis

Statistical analysis was performed in Graphpad Prism (version 8.0. RRID: SCR_002798) or R (version 4.3.3). The experimental data were presented as mean ± SEM. Unpaired two‐sided Student's *t* test or one‐way analysis of variance (ANOVA) was used to evaluate the significance of mean differences. Significance was indicated by * for *p* < 0.05, ** for *p* < 0.01, and *** for *p* < 0.001. SnRNA‐seq analysis was conducted using R language.

## Conflicts of Interest

The authors declare no conflicts of interest.

## Supporting information




**Supporting File**: advs74425‐sup‐0001‐SuppMat.docx.

## Data Availability

All data supporting the conclusions of this study are provided in the article and the Supporting Information. Raw data generated during the study are available from the corresponding authors upon reasonable request.
