# Supplementary material for: SR‐B1‐Mediated Transplacental Transfer of Hydrophobic Toxicants Disrupts Fetal Development During Barriergenesis
Source: Adv Sci (Weinh). 2026 Feb 25;13(23):e15742. doi: 10.1002/advs.202515742 (PMC13104103; doi:10.1002/advs.202515742)
Supplement: Supplementary file 1 — Supporting File: advs74425‐sup‐0001‐SuppMat.docx. [file ADVS-13-e15742-s001.docx]

Supporting Information

for

SR-B1-mediated Transplacental Transfer of Hydrophobic Toxicants Disrupts Fetal Development during Barriergenesis

Yixuan Huang^1#^, Hailin Shang^1#^, Ling Jiao^1^, Ce Chen^1^, Zehua Liu^1^, Yi Yang^1^, Qiannan Zhang^2^, Song Tang^3^, Xudong Jia^2^, Hui Yang^2^*, Yi Wan^1^*

Yixuan Huang, Hailin Shang, Ling Jiao, Ce Chen, Zehua Liu, Yi Yang, Yi Wan*
*Laboratory for Earth Surface Processes,* College of Urban and Environmental Sciences, Peking University, Beijing 100871, China
E-mail: wany@urban.pku.edu.cn

Qiannan Zhang, Xudong Jia, Hui Yang*
NHC Key Laboratory of Food Safety Risk Assessment, China National Center for Food Safety Risk Assessment, Beijing 100021, China
E-mail: yanghui@cfsa.net.cn

Song Tang
China CDC Key Laboratory of Environment and Population Health, Chinese Center for Disease Control and Prevention, Beijing 100021, China

# Yixuan Huang and Hailin Shang contributed equally to this work.

**Table S1.** Information of imageable MCCP congeners

| **Formula** | **Theoretical *m/z*** | **Measured *m/z*** | **Adduct** | **Error (ppm)** |
| --- | --- | --- | --- | --- |
| C_14_H_20_Cl_10_ | 576.8085 | 576.8093 | [M + Cl]^-^ | -1.39 |
| C_14_H_21_Cl_9_ | 542.8475 | 542.8479 | [M + Cl]^-^ | -0.74 |
| C_14_H_22_Cl_8_ | 508.8864 | 508.887 | [M + Cl]^-^ | -1.18 |
| C_14_H_23_Cl_7_ | 474.9254 | 474.9258 | [M + Cl]^-^ | -0.84 |
| C_14_H_24_Cl_6_ | 438.9673 | 438.9685 | [M + Cl]^-^ | -2.73 |
| C_14_H_25_Cl_5_ | 405.0063 | 405.0068 | [M + Cl]^-^ | -1.23 |
| C_14_H_26_Cl_4_ | 371.0453 | 371.0457 | [M + Cl]^-^ | -1.08 |
| C_15_H_22_Cl_10_ | 590.8241 | 590.8253 | [M + Cl]^-^ | -2.03 |
| C_15_H_23_Cl_9_ | 556.8631 | 556.8635 | [M + Cl]^-^ | -0.72 |
| C_15_H_24_Cl_8_ | 522.9021 | 522.9026 | [M + Cl]^-^ | -0.96 |
| C_15_H_25_Cl_7_ | 488.9410 | 488.9416 | [M + Cl]^-^ | -1.23 |
| C_15_H_26_Cl_6_ | 452.9830 | 452.9837 | [M + Cl]^-^ | -1.55 |
| C_16_H_23_Cl_11_ | 640.7979 | 640.7990 | [M + Cl]^-^ | -1.72 |
| C_16_H_24_Cl_10_ | 604.8398 | 604.8407 | [M + Cl]^-^ | -1.49 |
| C_16_H_25_Cl_9_ | 570.8788 | 570.8795 | [M + Cl]^-^ | -1.23 |
| C_16_H_26_Cl_8_ | 536.9177 | 536.9183 | [M + Cl]^-^ | -1.12 |
| C_16_H_27_Cl_7_ | 502.9567 | 502.9575 | [M + Cl]^-^ | -1.59 |
| C_16_H_28_Cl_6_ | 466.9986 | 466.9990 | [M + Cl]^-^ | -0.86 |
| C_17_H_25_Cl_11_ | 654.8135 | 654.8146 | [M + Cl]^-^ | -1.68 |
| C_17_H_26_Cl_10_ | 618.8554 | 618.8564 | [M + Cl]^-^ | -1.62 |
| C_17_H_27_Cl_9_ | 584.8944 | 584.8952 | [M + Cl]^-^ | -1.37 |
| C_17_H_28_Cl_8_ | 550.9334 | 550.9341 | [M + Cl]^-^ | -1.27 |
| C_17_H_29_Cl_7_ | 516.9723 | 516.9728 | [M + Cl]^-^ | -0.97 |
| C_17_H_30_Cl_6_ | 481.0143 | 481.0148 | [M + Cl]^-^ | -1.04 |

**Table S2.** The parameters of MRM transitions, cone voltages, and collision energies

| Chemicals | Precursor Ion (*m/z*) | Product Ion (*m/z*) | Cone Voltages (V) | Collision Energies (eV) |
| --- | --- | --- | --- | --- |
| AMX | 366.4 | 160.1 | 15 | 19 |
|  |  | 114.0* |  | 29 |
| AMP | 350.4 | 192.1 | 20 | 18 |
|  |  | 160.1* |  | 23 |
| MEHP | 277.1 | 133.9* | 6 | 14 |
|  |  | 127.1 |  | 6 |

*Quantitative ions.


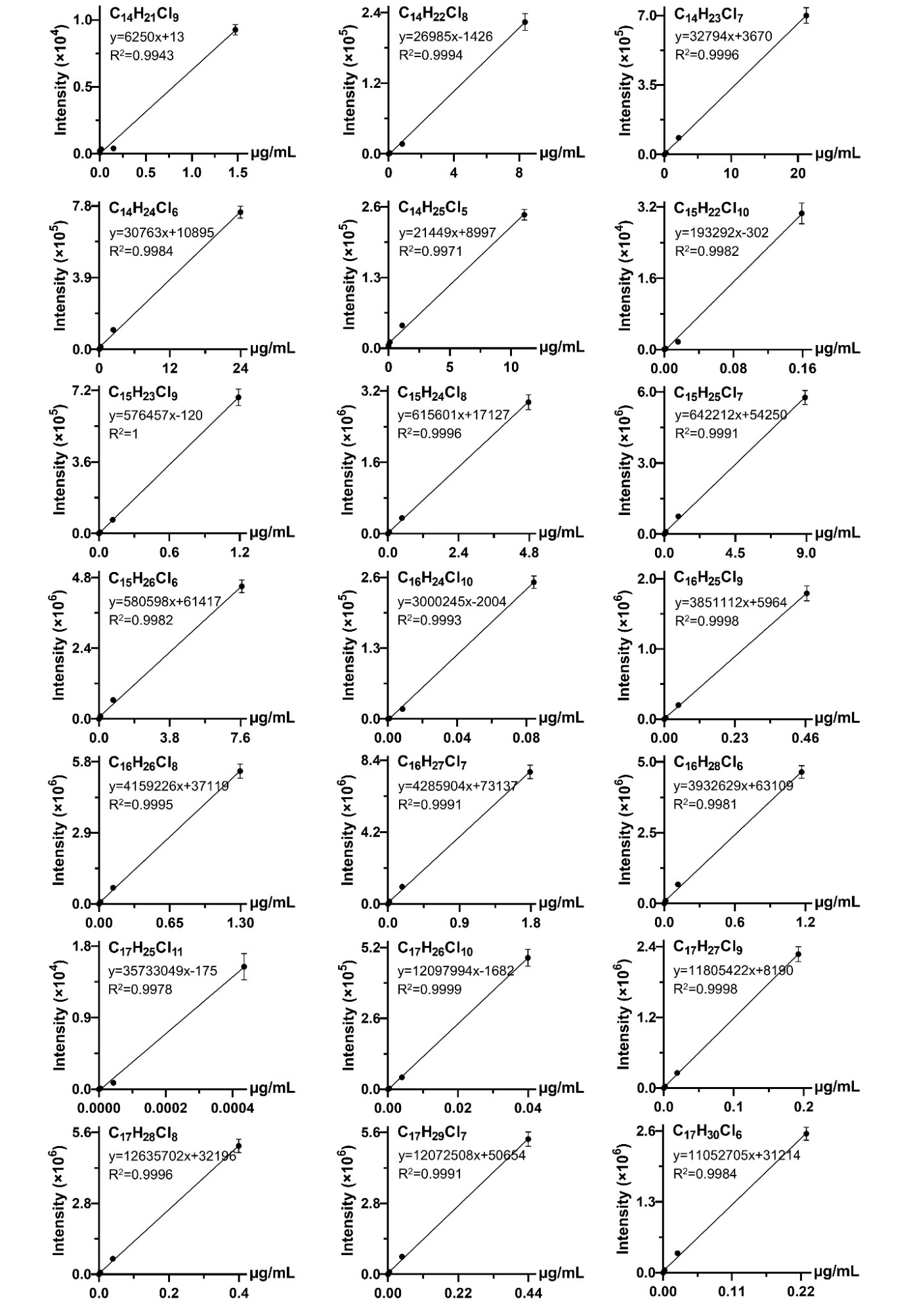


**Figure S1.** Calibration curves of MCCP congeners using the established imaging method. Three replicates per concentration point were analyzed. Data represent mean ± SEM. MCCPs, medium-chain chlorinated paraffins.

**
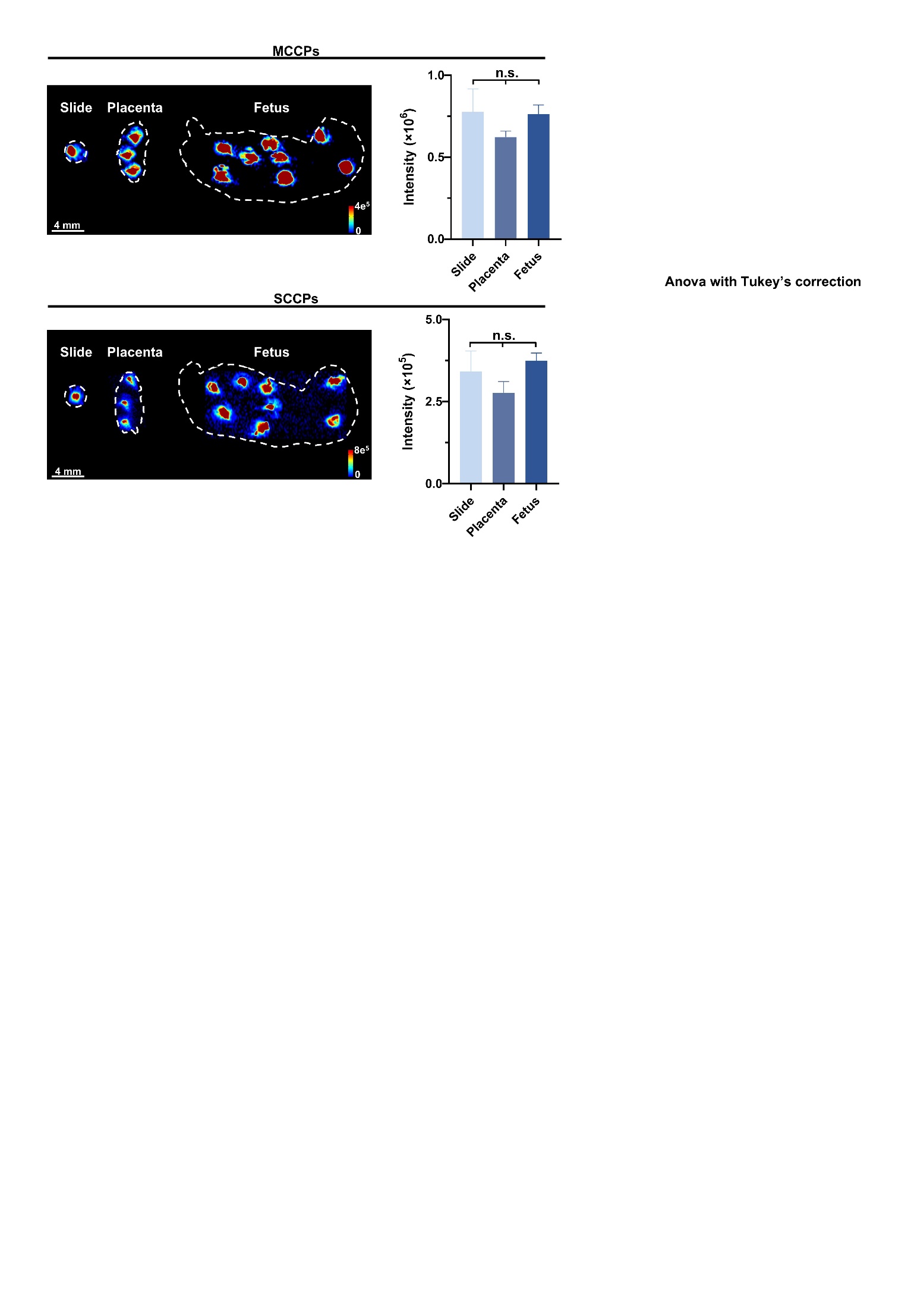
**

**Figure S2.** Evaluation of matrix effects of imaging analysis of MCCPs and SCCPs using Ph_4_PCl-enhanced AFAI-MSI (*n* = 3 replicate spots in blank section; *n* = 3 replicate spots in unexposed placenta; *n* = 8 replicate spots in unexposed fetus). Data represent mean ± SEM. n.s., not significant; **p* < 0.05, ***p* < 0.01, ****p* < 0.001. MCCPs, medium-chain chlorinated paraffins; SCCPs, short-chain chlorinated paraffins; Ph_4_PCl, tetraphenylphosphonium chloride; AFAI-MSI, air flow-assisted ionization-mass spectrometry imaging.


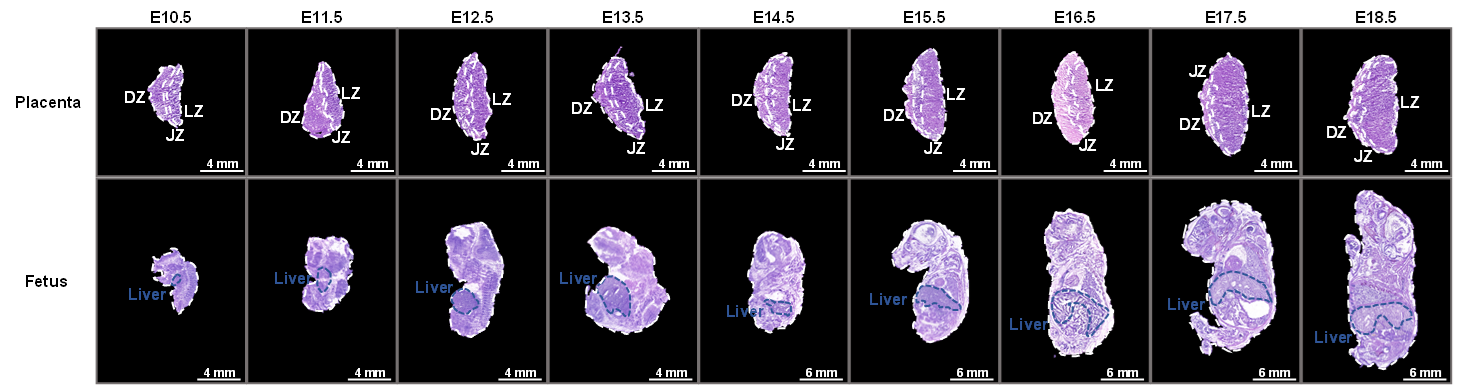


**Figure S3.** Representative H&E-stained sections of murine placenta and fetus at each development timepoint. DZ, decidual zone; JZ, junctional zone; LZ, labyrinth zone.


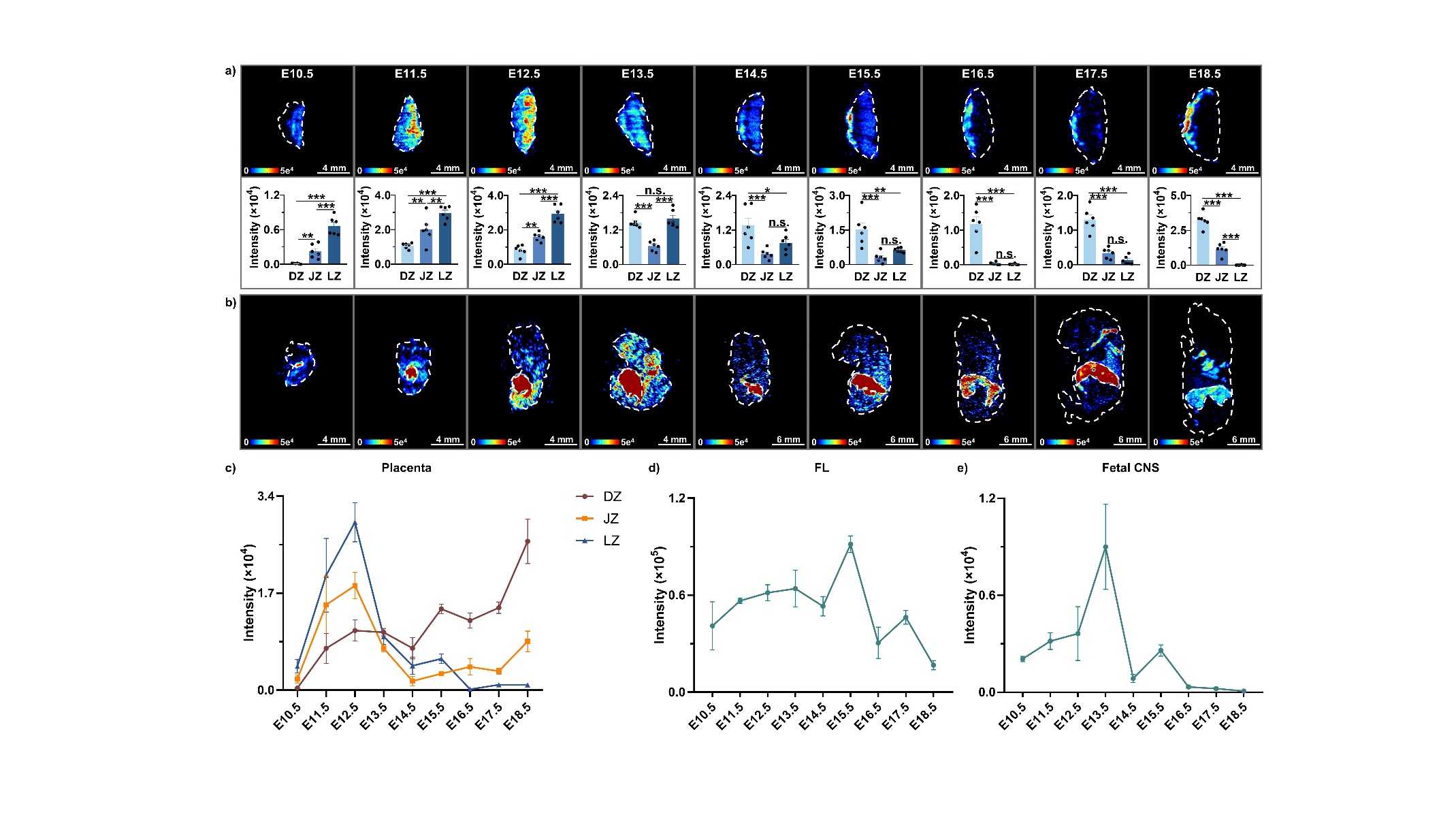


**Figure S4.** Spatiotemporal heterogeneity of hydrophobic MCCPs (representative congener: C_15_H_24_Cl_8_) in the developing placenta and fetus. (a) MS images and intensity of MCCPs in the developing placenta (*n* = 6 independent section regions were selected across three biological replicates). (b) Spatial distribution of MCCPs in the developing fetus. (c-e) Dynamic trends of MCCP intensity in distinct placental regions (c), the FL (d) and the fetal CNS (e) (three biological replicates per development timepoint). Data represent mean ± SEM. n.s., not significant; **p* < 0.05, ***p* < 0.01, ****p* < 0.001. MCCPs, medium-chain chlorinated paraffins; DZ, decidual zone; JZ, junctional zone; LZ, labyrinth zone; FL, fetal liver; CNS, central nervous system.


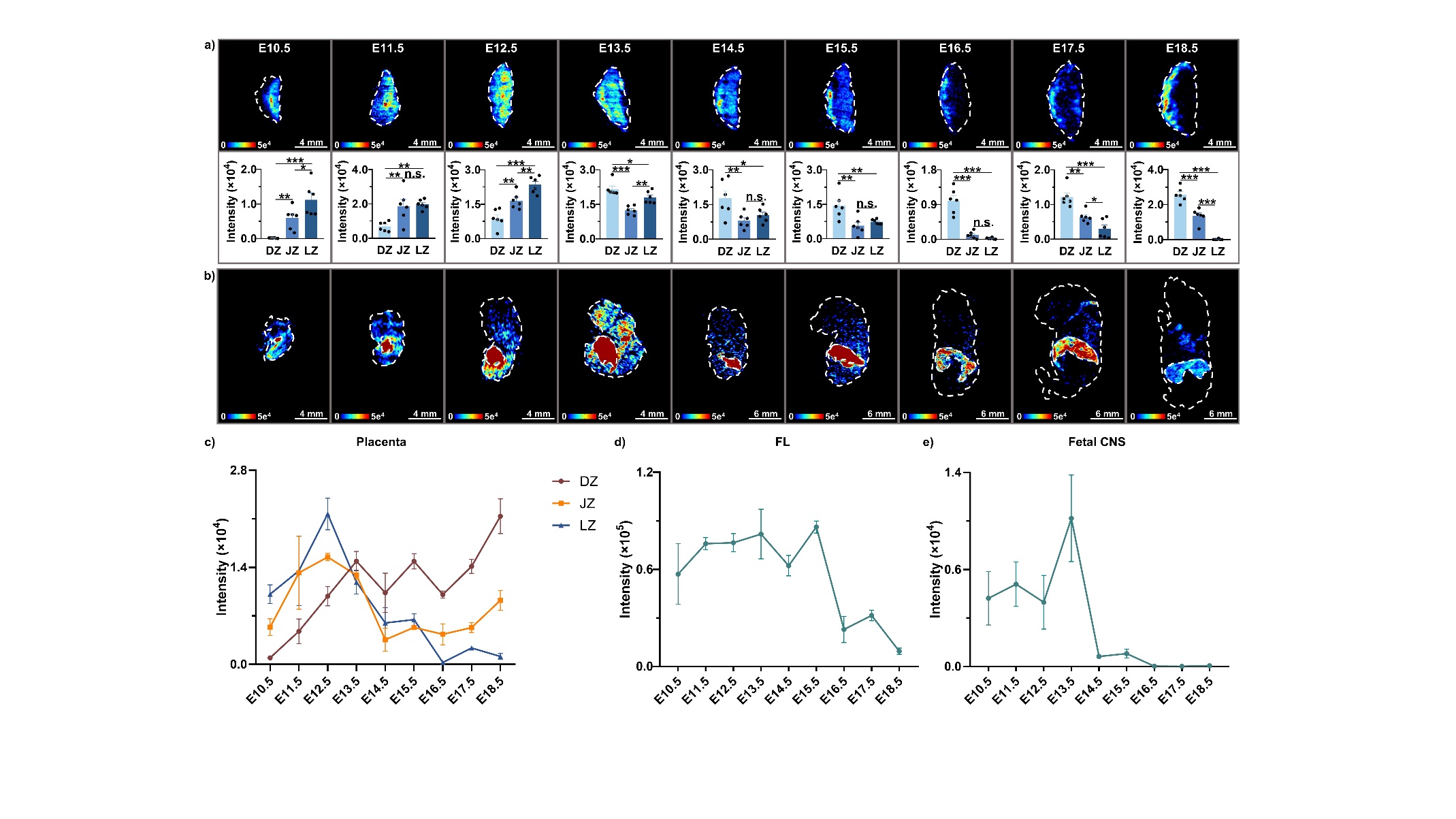


**Figure S5.** Spatiotemporal heterogeneity of hydrophobic MCCPs (representative congener: C_17_H_29_Cl_7_) in the developing placenta and fetus. (a) MS images and intensity of MCCPs in the developing placenta (*n* = 6 independent section regions were selected across three biological replicates). (b) Spatial distribution of MCCPs in the developing fetus. (c-e) Dynamic trends of MCCP intensity in distinct placental regions (c), the FL (d) and the fetal CNS (e) (three biological replicates per development timepoint). Data represent mean ± SEM. n.s., not significant; **p* < 0.05, ***p* < 0.01, ****p* < 0.001. MCCPs, medium-chain chlorinated paraffins; DZ, decidual zone; JZ, junctional zone; LZ, labyrinth zone; FL, fetal liver; CNS, central nervous system.


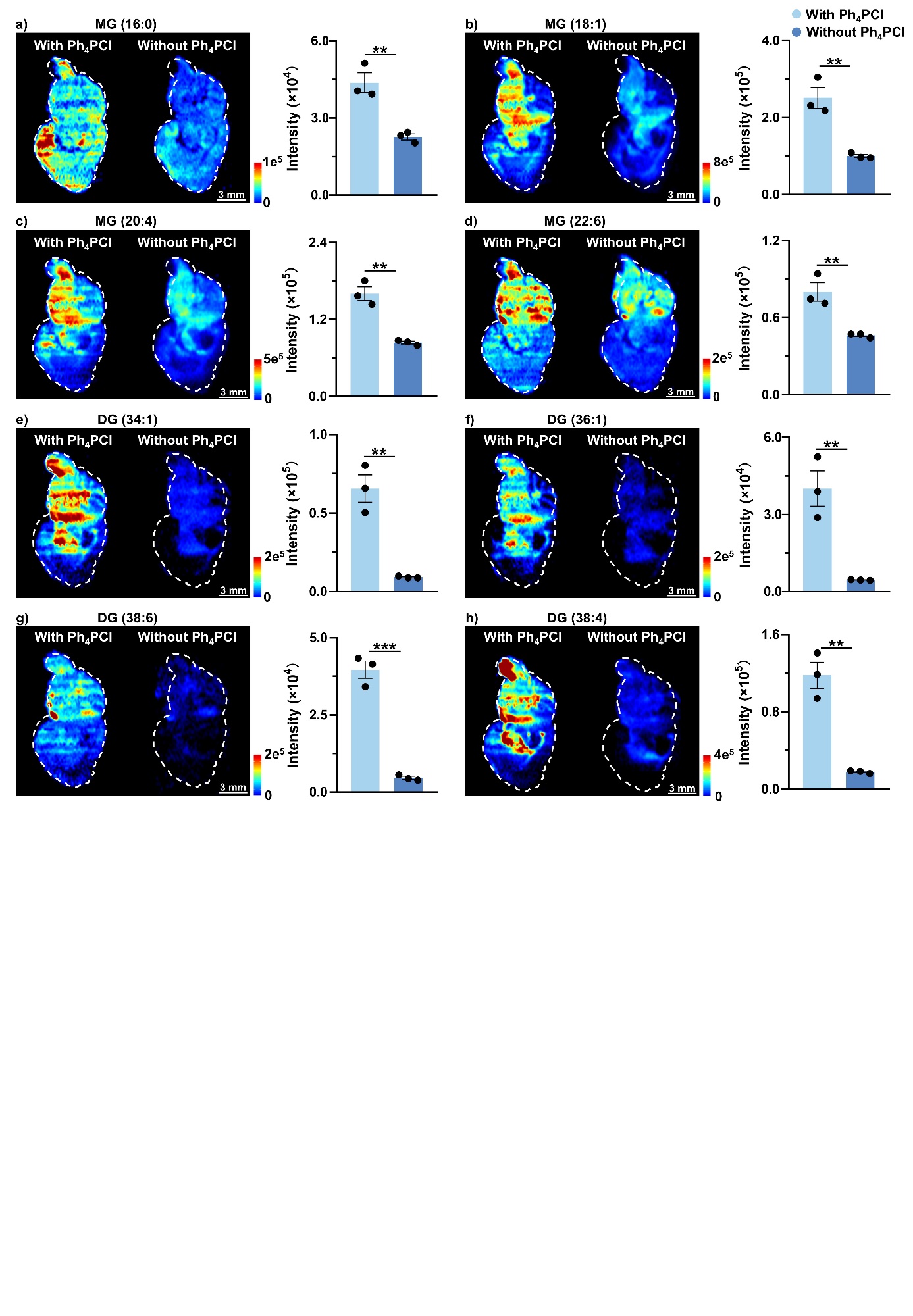


**Figure S6.** Ph_4_PCl-enhanced ionization improved the analytical sensitivities of MG (a-d) and DG (e-h). (*n* = 3 biological replicates per group). Data represent mean ± SEM. **p* < 0.05, ***p* < 0.01, ****p* < 0.001. MG, monoacylglycerol; DG, diacylglycerol; Ph_4_PCl, tetraphenylphosphonium chloride.


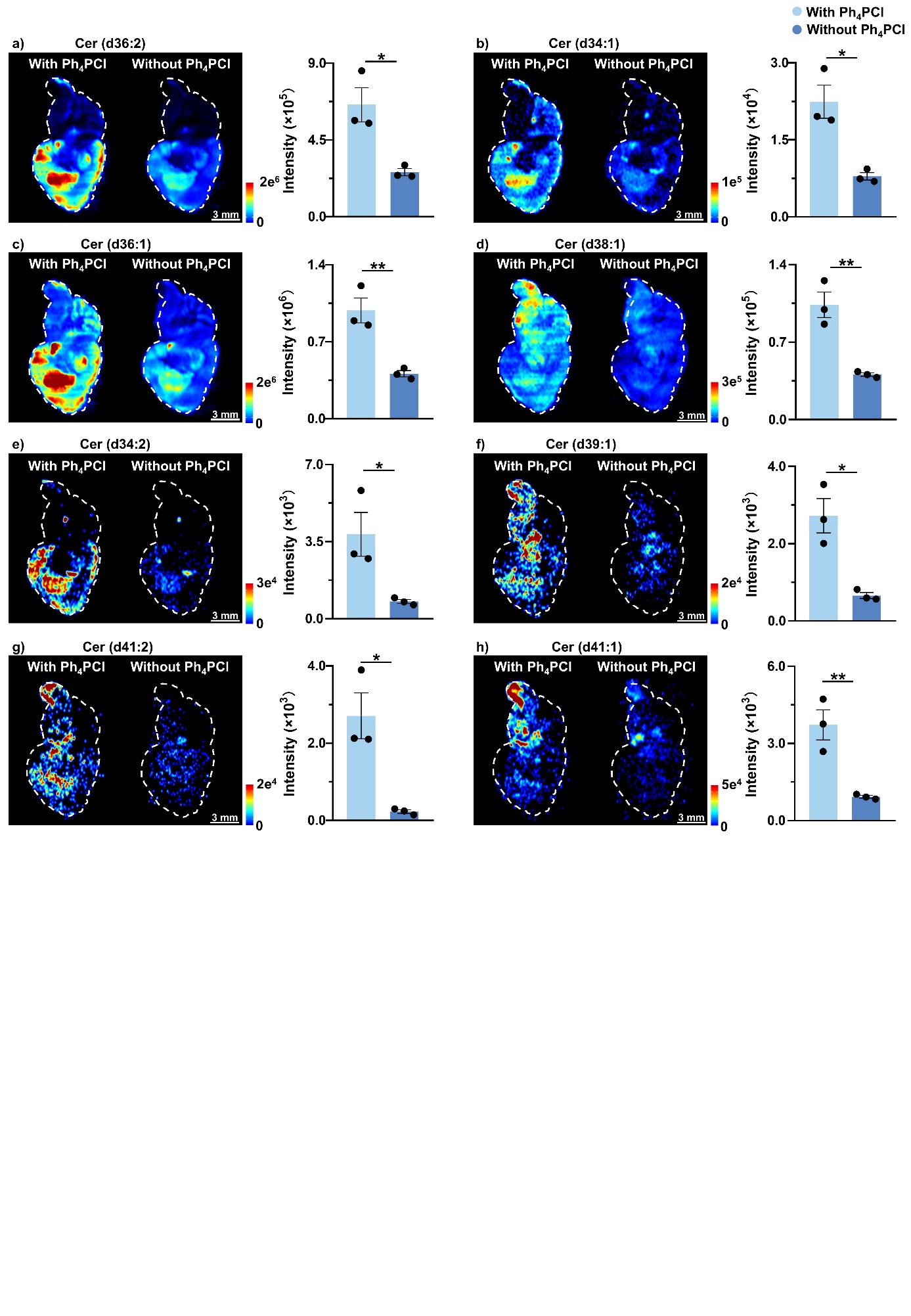


**Figure S7.** Ph_4_PCl-enhanced ionization improved the analytical sensitivities of ceramides. (*n* = 3 biological replicates per group). Data represent mean ± SEM. **p* < 0.05, ***p* < 0.01, ****p* < 0.001. Cer, ceramide; Ph_4_PCl, tetraphenylphosphonium chloride.

**Figure S8.** Spatiotemporal heterogeneity of representative endogenous lipids in the developing placenta. PE, phosphatidylethanolamine; DG, diacylglycerol; FA, fatty acid.

**Figure S9.** MS images and intensity of endogenous metabolites in the placenta at E11.5 across treatment groups. *n* = 6 independent section regions were selected across four biological replicates. Data represent mean ± SEM. n.s., not significant; **p* < 0.05, ***p* < 0.01, ****p* < 0.001. DZ, decidual zone; JZ, junctional zone; LZ, labyrinth zone; PE, phosphatidylethanolamine; TG, triglyceride; FA, fatty acid; Cer, ceramide; PC, phosphatidylcholine; DG, diacylglycerol; MG, monoglyceride; PG, phosphatidylglycerol; PI, phosphatidylinositol; 5-HK, 5-Hydroxy kynurenine; 2-HG, 2-Hydroxyglutarate; HOG, 4-Hydroxy-2-oxoglutarate.

**Figure S10.** MS images and intensity of endogenous metabolites in the placenta at E17.5 across MCCP exposed groups. *n* = 6 independent section regions were selected across four biological replicates. Data represent mean ± SEM. n.s., not significant; **p* < 0.05, ***p* < 0.01, ****p* < 0.001. DZ, decidual zone; JZ, junctional zone; LZ, labyrinth zone; PE, phosphatidylethanolamine; TG, triglyceride; FA, fatty acid; Cer, ceramide; PG, phosphatidylglycerol; PS, phosphatidylserine; PA, phosphatidic acid; SM, sphingomyelin; LysoPA, lysophosphatidic acid; EODA, 9,10-epoxyoctadecanoic acid; 2-OSM, 2-oxosuccinamate; HCA, homocitric acid; IAA-R, imidazoleacetic acid riboside; NAT, N-acetyl-L-tyrosine; ACC, 1-aminocyclopropanecarboxylic acid; DITP, 2’-deoxyinosine triphosphate; PCA, pyroglutamic acid; 2-AMS, 2-aminomuconate semialdehyde.


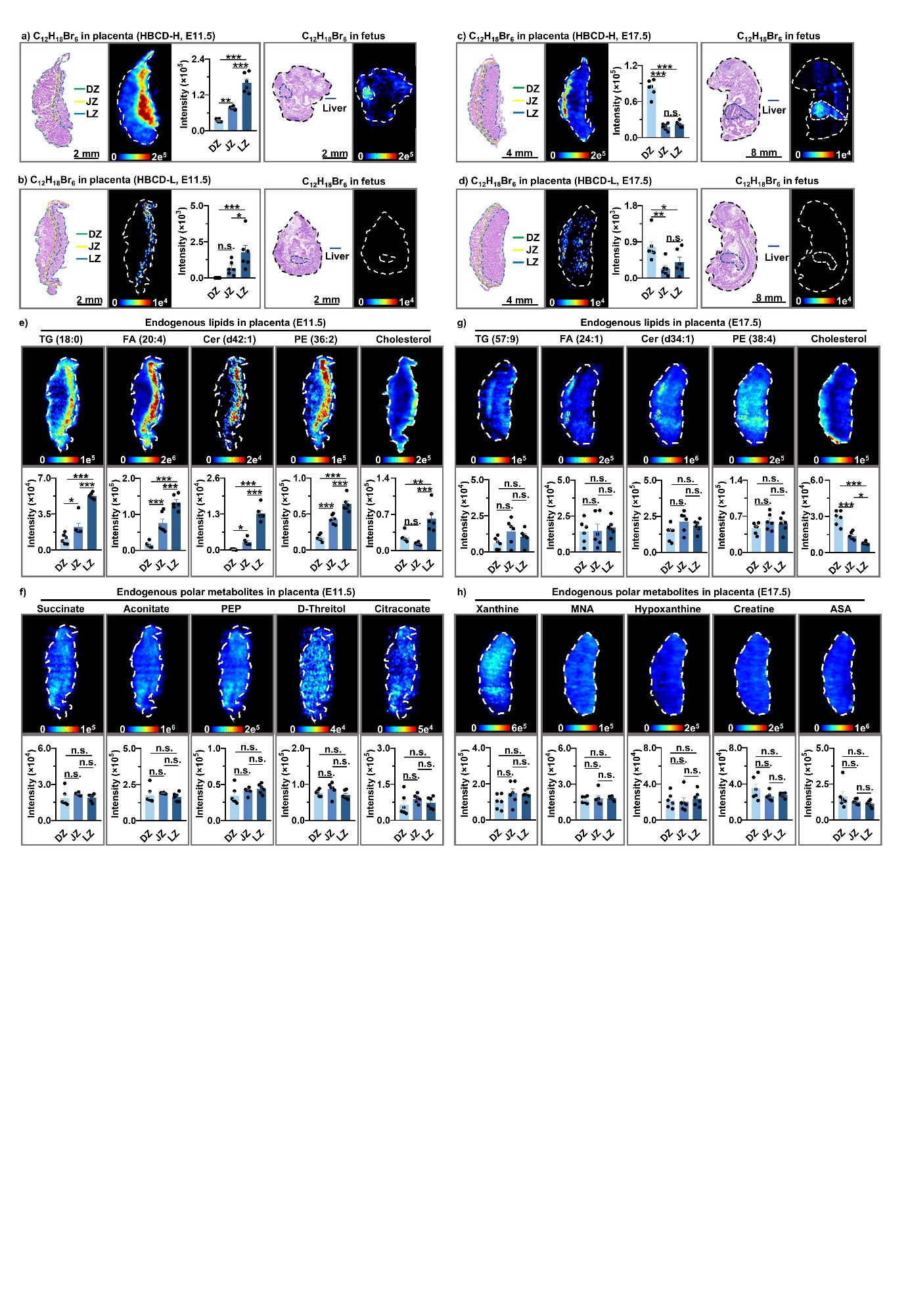


**Figure S11.** Spatial distributions of hydrophobic HBCD and endogenous lipids in the placenta at E11.5 and E17.5. (a-d) H&E-stained sections, MS images and intensity of HBCD in the placenta and fetus at E11.5 in the HBCD-H group (a), HBCD-L group (b), at E17.5 in the HBCD-H group (c), and HBCD-L group (d). (e-h) MS images and intensity of endogenous lipids and polar metabolites in the placenta at E11.5 in the HBCD-L group (e-f) and at E17.5 in the HBCD-L group (g-h). *n* = 6 independent section regions were selected across four biological replicates. Data represent mean ± SEM. n.s., not significant; **p* < 0.05, ***p* < 0.01, ****p* < 0.001. HBCD, hexabromocyclododecane; DZ, decidual zone; JZ, junctional zone; LZ, labyrinth zone; TG, triglyceride; FA, fatty acid; Cer, ceramide; PE, phosphatidylethanolamine; PEP, phosphoenolpyruvic acid; MNA, 1-methylnicotinamide; ASA, argininosuccinic acid.


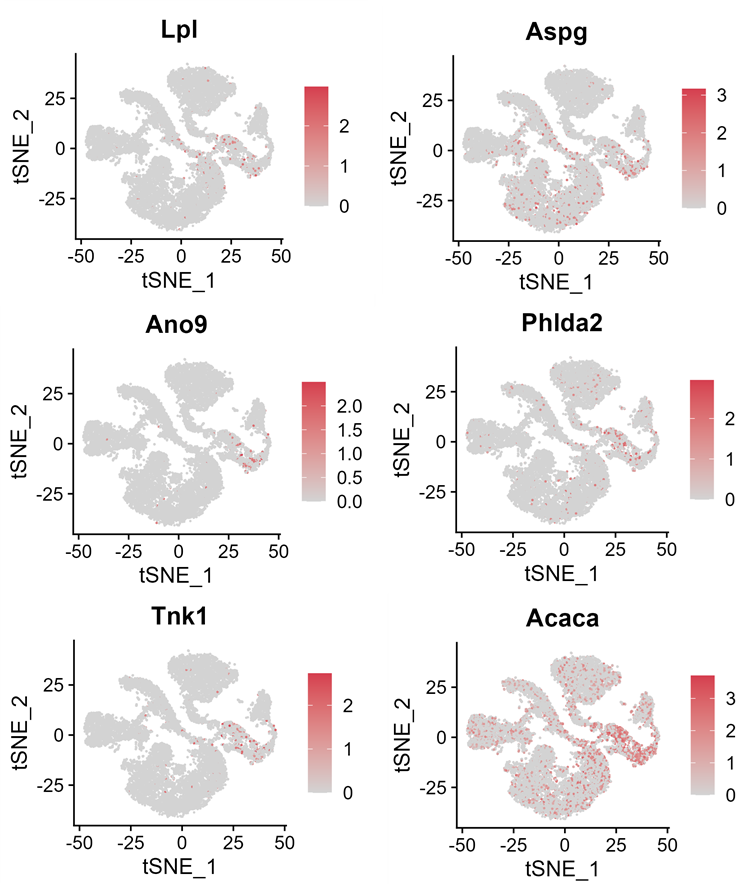


**Figure S12.** Expression of screened protein candidates visualized on t-SNE projections. t-SNE, t-distributed stochastic neighbor embedding; Lpl, Lipoproteinlipase; Aspg, Asparaginase; Ano9, Anoctamin 9; Phlda2, Pleckstrin homology like domain family A member 2; Tnk1, Tyrosine kinase non receptor 1; Acaca, Acetyl-coA carboxylase alpha.


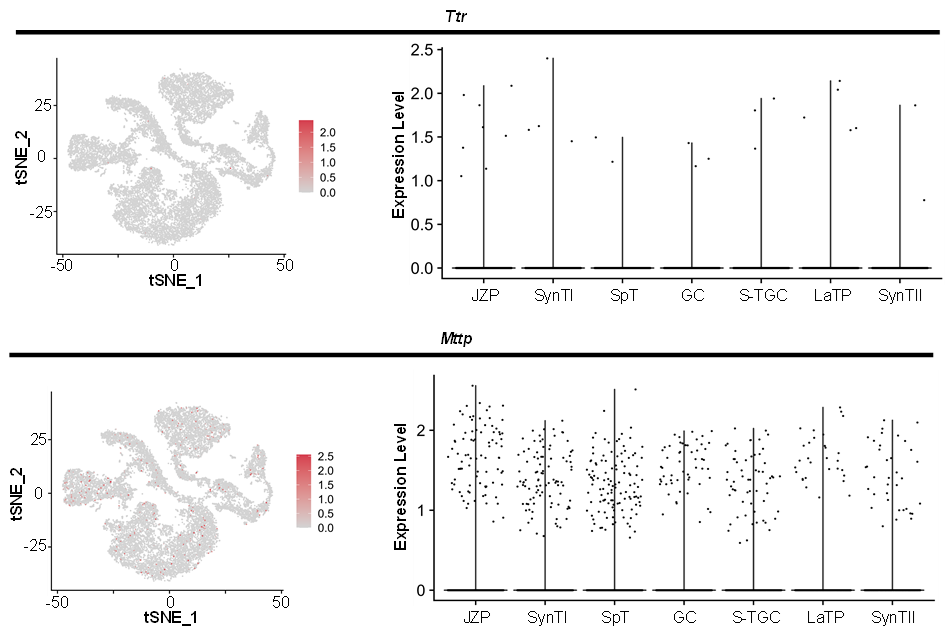


**Figure S13.** *Ttr* and *Mttp* expression visualized on t-SNE projections and summarized as a violin plot. t-SNE, t-distributed stochastic neighbor embedding; Ttr, Transthyretin; Mttp, Microsomal triglyceride transfer protein.


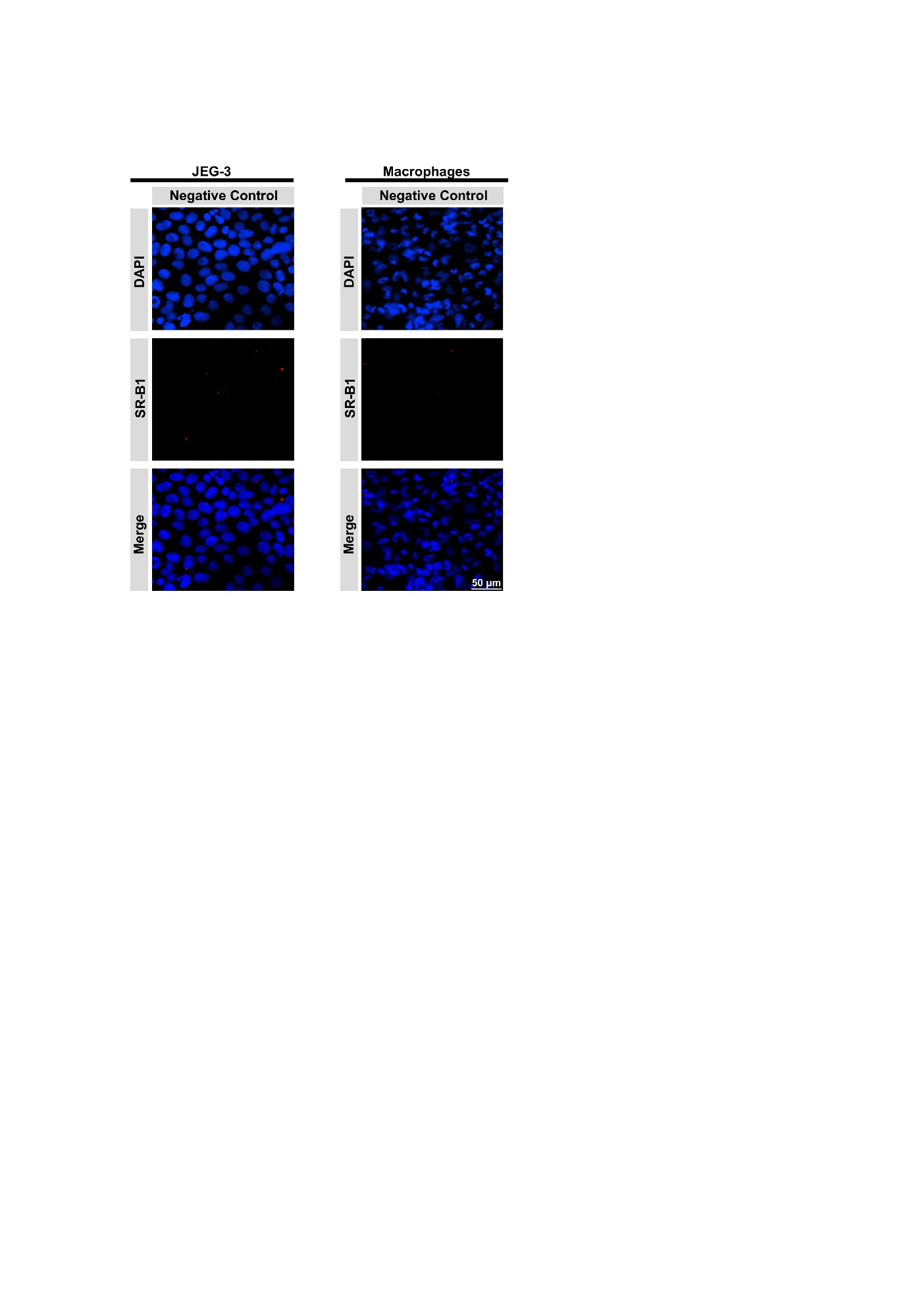


**Figure S14.** Negative control immunofluorescence images of SR-B1 in JEG-3 and macrophages. SR-B1, Scavenger Receptor Class B Member 1.


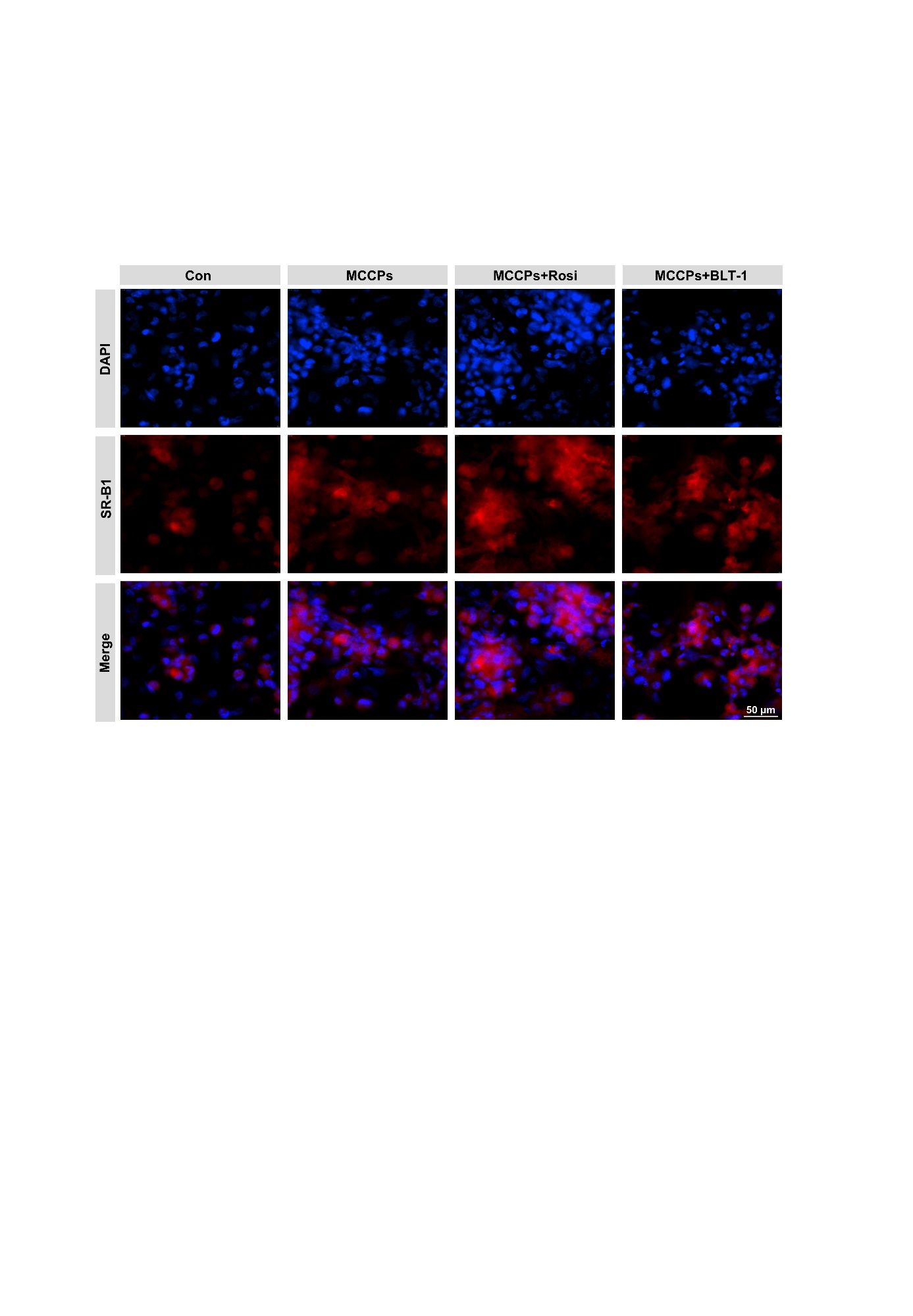


**Figure S15.** Representative images of SR-B1 expression in macrophages across treatment groups. MCCPs, medium-chain chlorinated paraffins; Rosi, rosiglitazone; BLT-1, block lipid transport-1; SR-B1, Scavenger Receptor Class B Member 1.


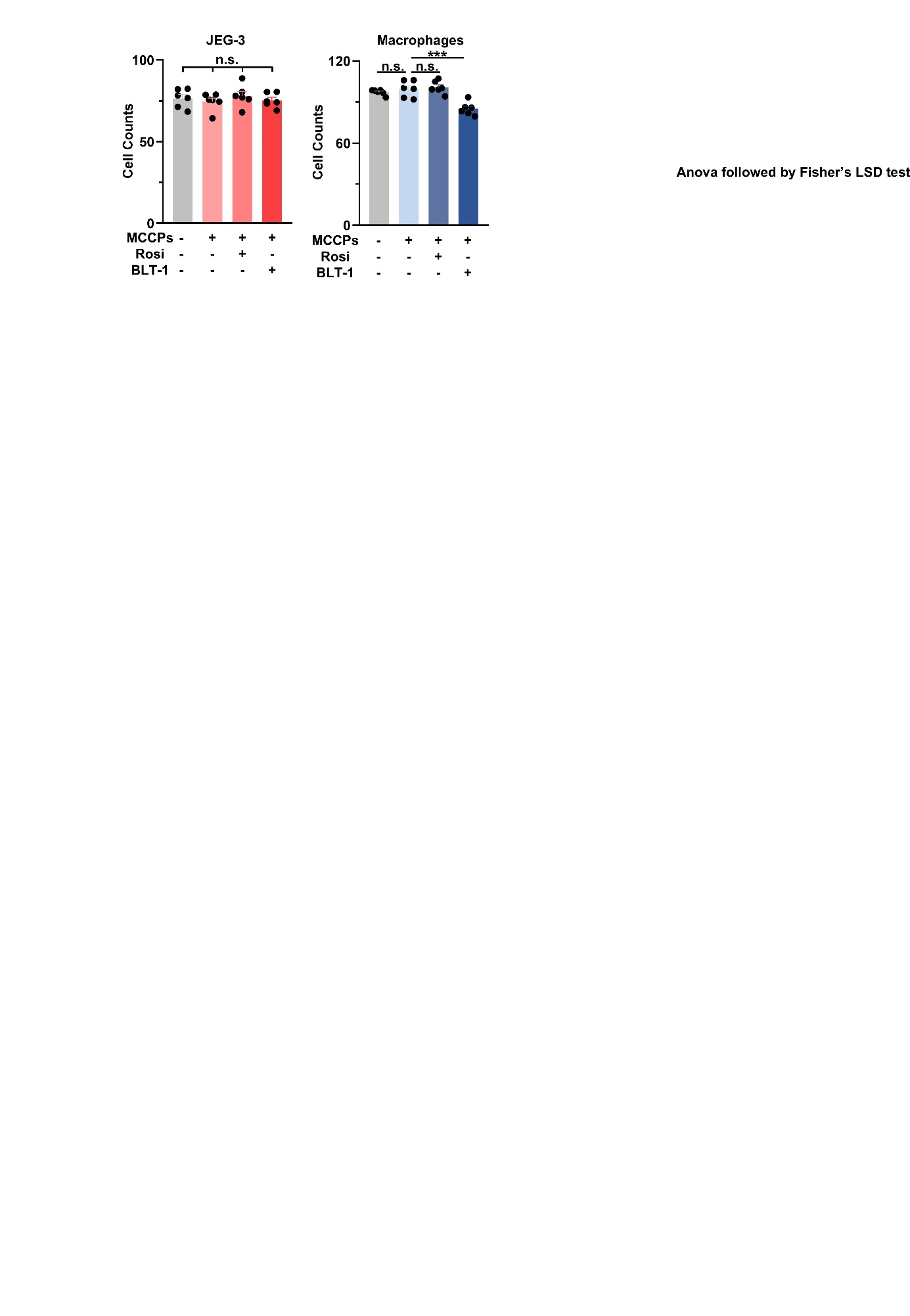


**Figure S16.** Cell counts of JEG-3 and macrophages across treatment groups (*n* = 6 biological replicates per group). Mild cytotoxicity (<15%) observed in macrophages co-treated with MCCPs and BLT-1. Data represent mean ± SEM. n.s., not significant; **p* < 0.05, ***p* < 0.01, ****p* < 0.001. MCCPs, medium-chain chlorinated paraffins; Rosi, rosiglitazone; BLT-1, block lipid transport-1.


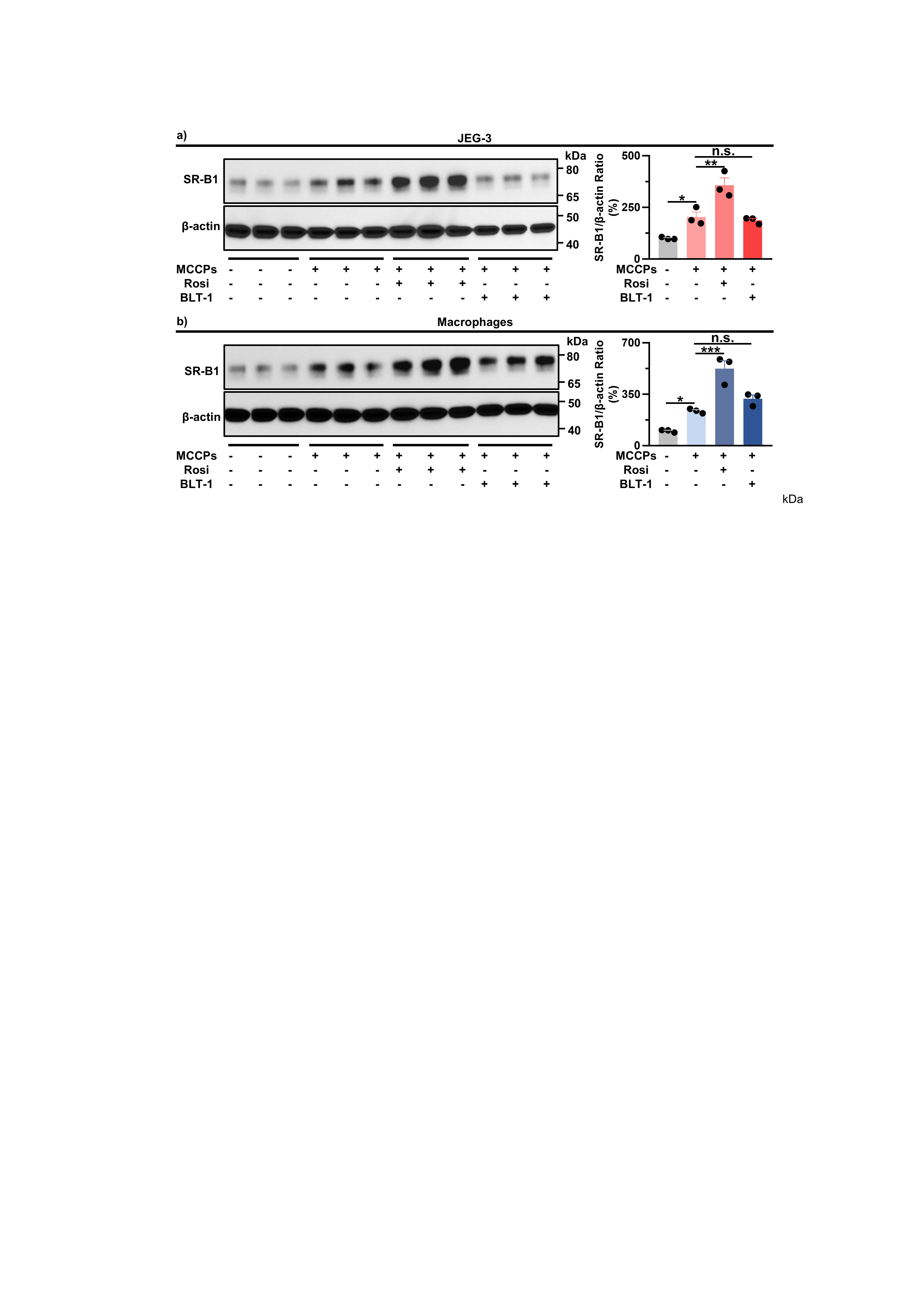


**Figure S17.** Western blots of SR-B1 expression in JEG-3 cells (a) and macrophages (b) (*n* = 3 biological replicates per group). Data represent mean ± SEM. n.s., not significant; **p* < 0.05, ***p* < 0.01, ****p* < 0.001. MCCPs, medium-chain chlorinated paraffins; Rosi, rosiglitazone; BLT-1, block lipid transport-1; SR-B1, Scavenger Receptor Class B Member 1.


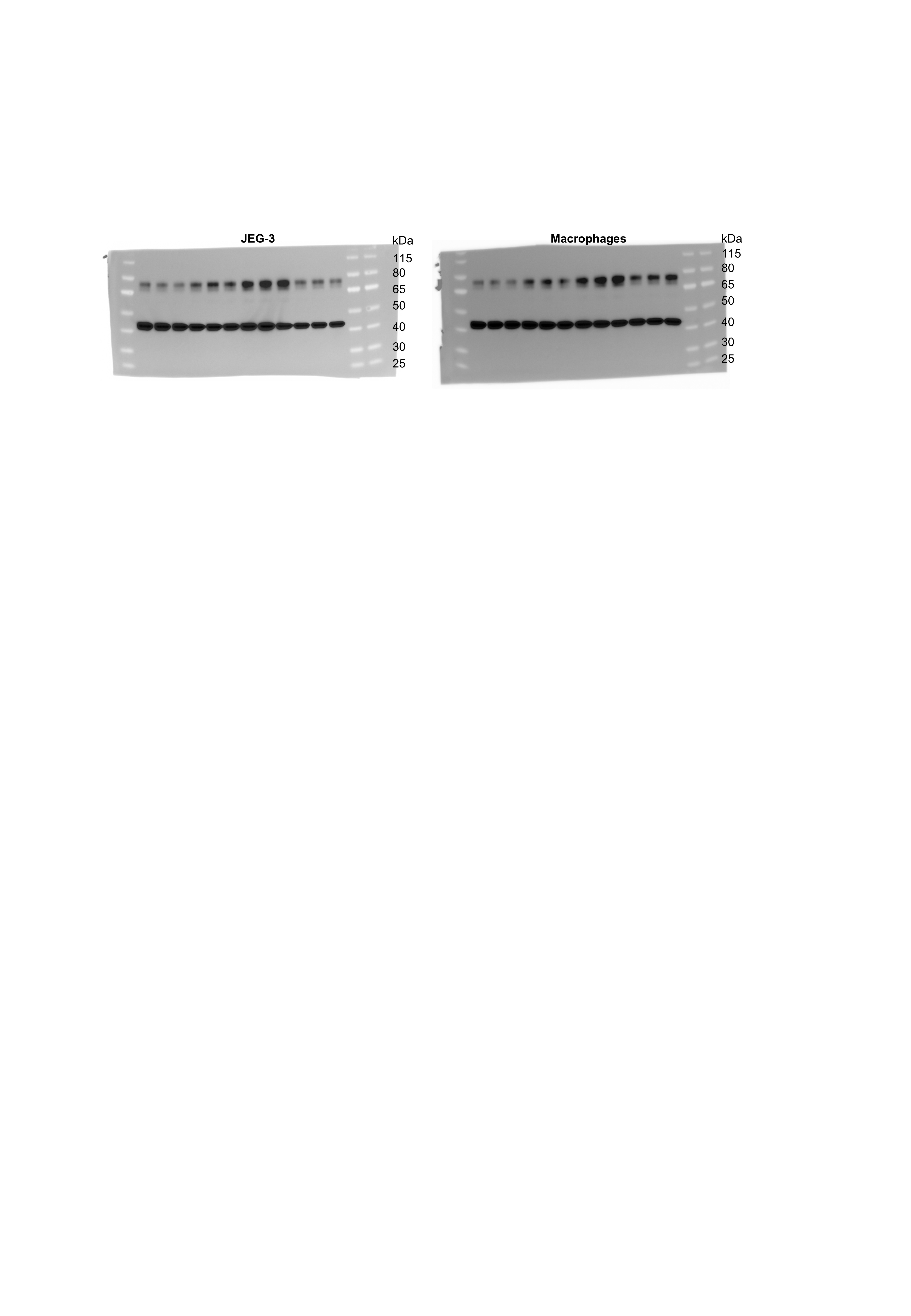


**Figure S18.** Uncropped blots of SR-B1 expression in JEG-3 cells and macrophages.


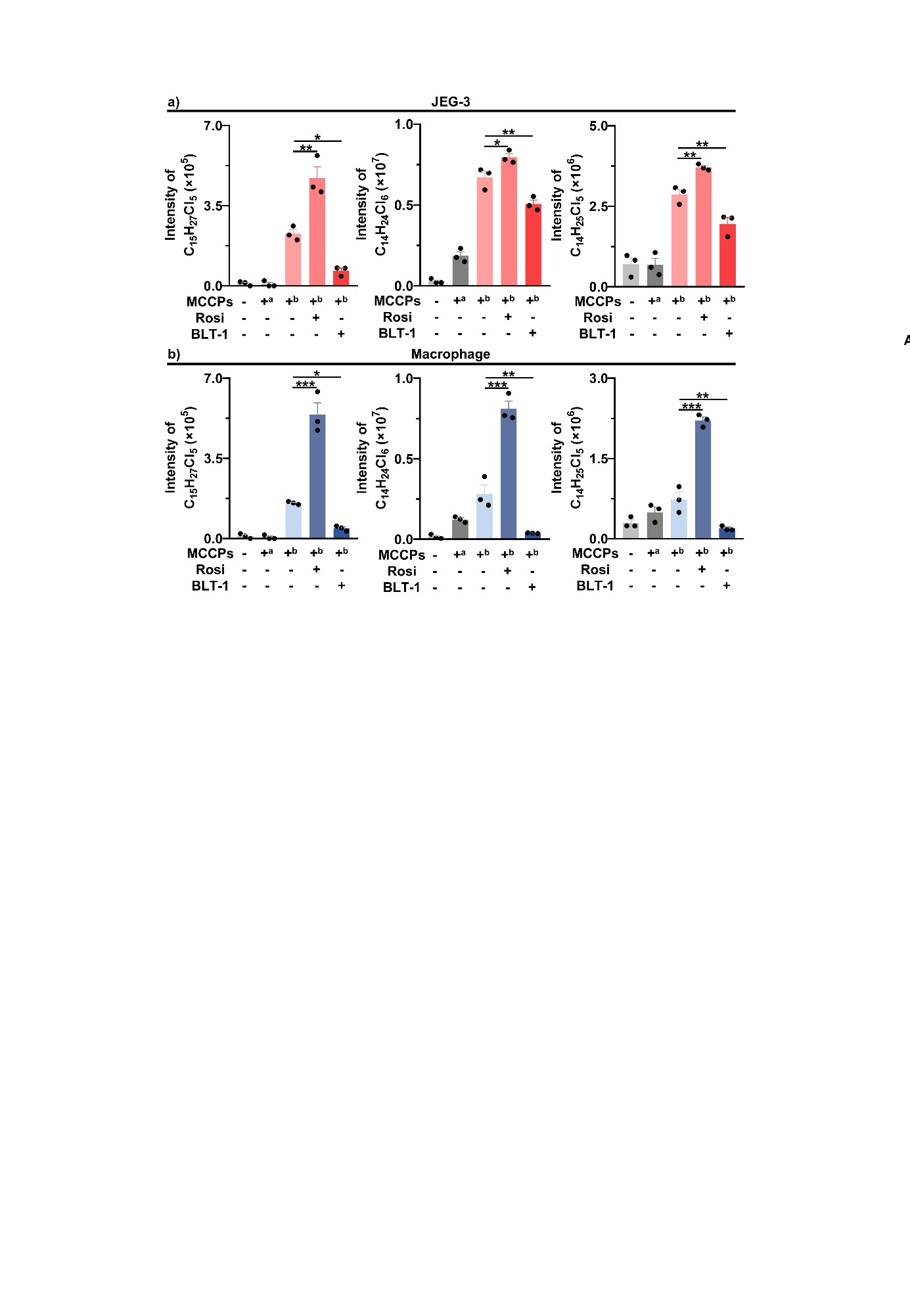


**Figure S19.** Intracellular MCCPs intensity in JEG-3 cells (a) and macrophages (b) across treatment groups (*n* = 3 biological replicates per group). a, exposure for 10 min; b, exposure for 72 h. Data represent mean ± SEM. **p* < 0.05, ***p* < 0.01, ****p* < 0.001. MCCPs, medium-chain chlorinated paraffins; Rosi, rosiglitazone; BLT-1, block lipid transport-1.


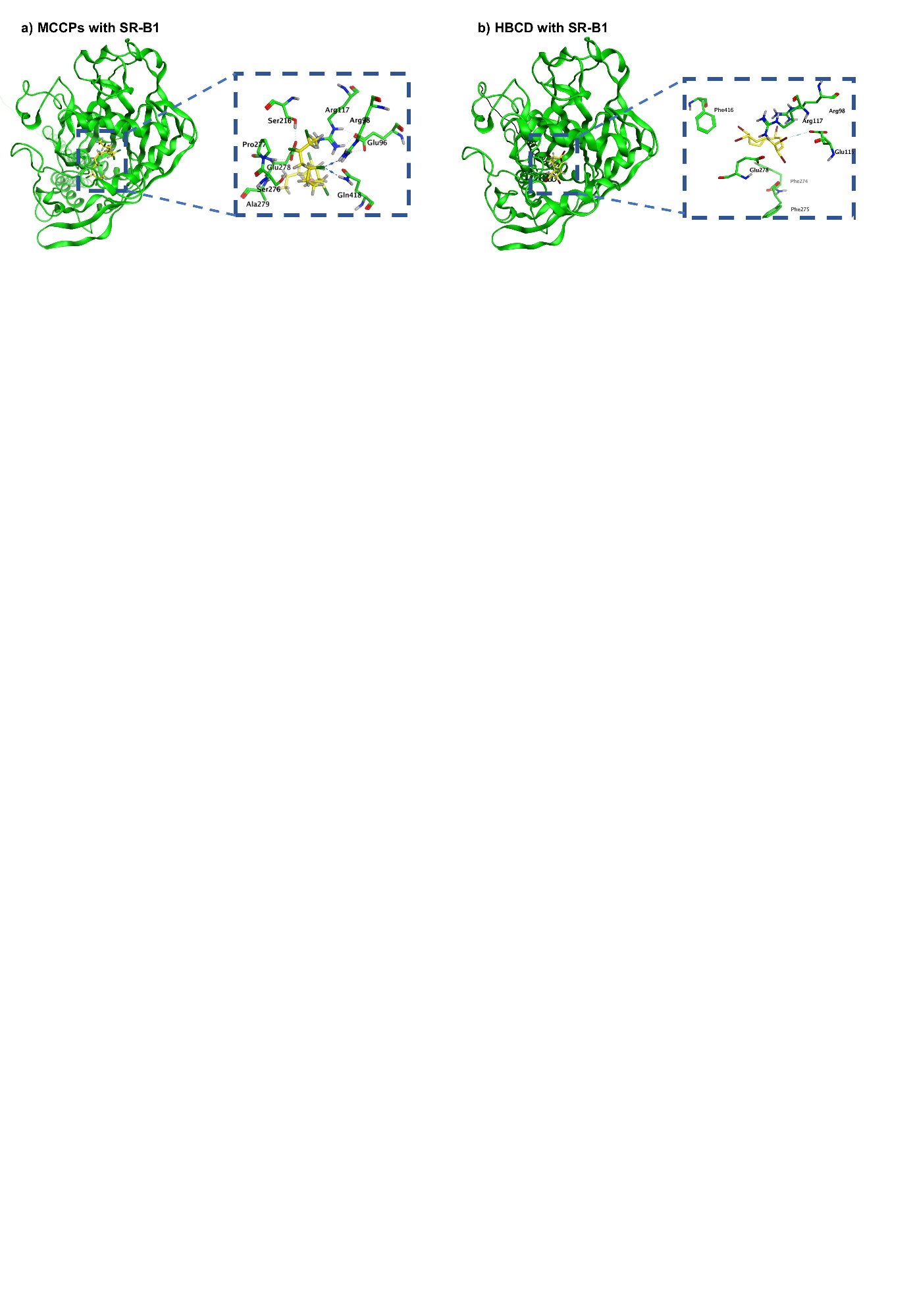


**Figure S20.** Molecular docking analysis of MCCPs with SR-B1 (a) and HBCD with SR-B1 (b). MCCPs, medium-chain chlorinated paraffins; HBCD, hexabromocyclododecane; SR-B1, Scavenger receptor class B member 1.


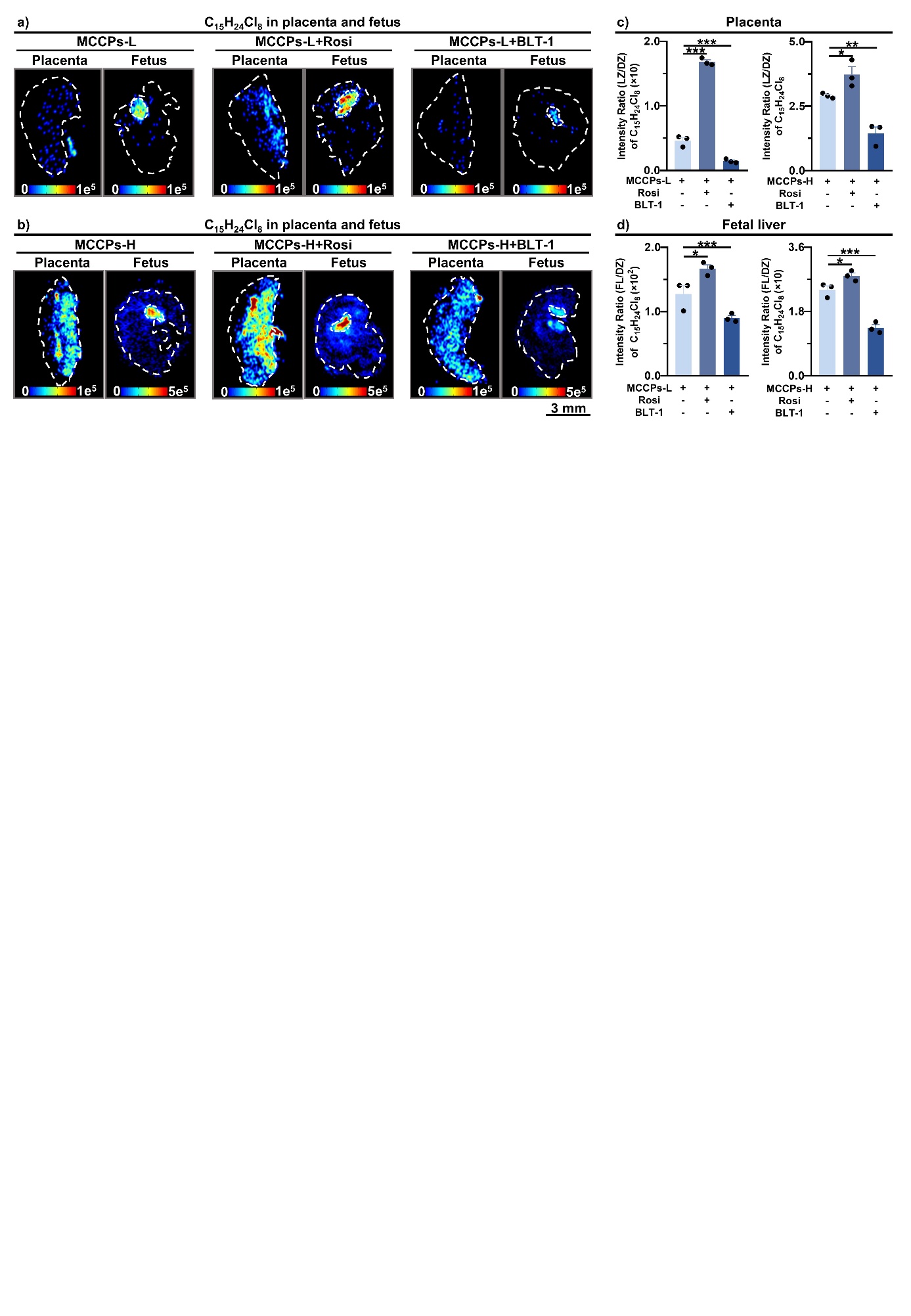


**Figure S21.** SR-B1-mediated transplacental transfer of hydrophobic MCCPs (representative congener: C_15_H_24_Cl_8_) *in vivo*. (a-b) MS images of MCCPs in the placenta and fetus at E11.5 across MCCP exposed groups. (c) Intensity ratio (LZ/DZ) of MCCPs in the placenta at E11.5 across MCCP exposed groups (*n* = 3 biological replicates per group). (d) Intensity ratio (FL/DZ) of MCCPs in FL at E11.5 across MCCP exposed groups (*n* = 3 biological replicates per group). Data represent mean ± SEM. **p* < 0.05, ***p* < 0.01, ****p* < 0.001. MCCPs, medium-chain chlorinated paraffins; Rosi, rosiglitazone; BLT-1, block lipid transport-1; DZ, decidual zone; LZ, labyrinth zone; FL, fetal liver.


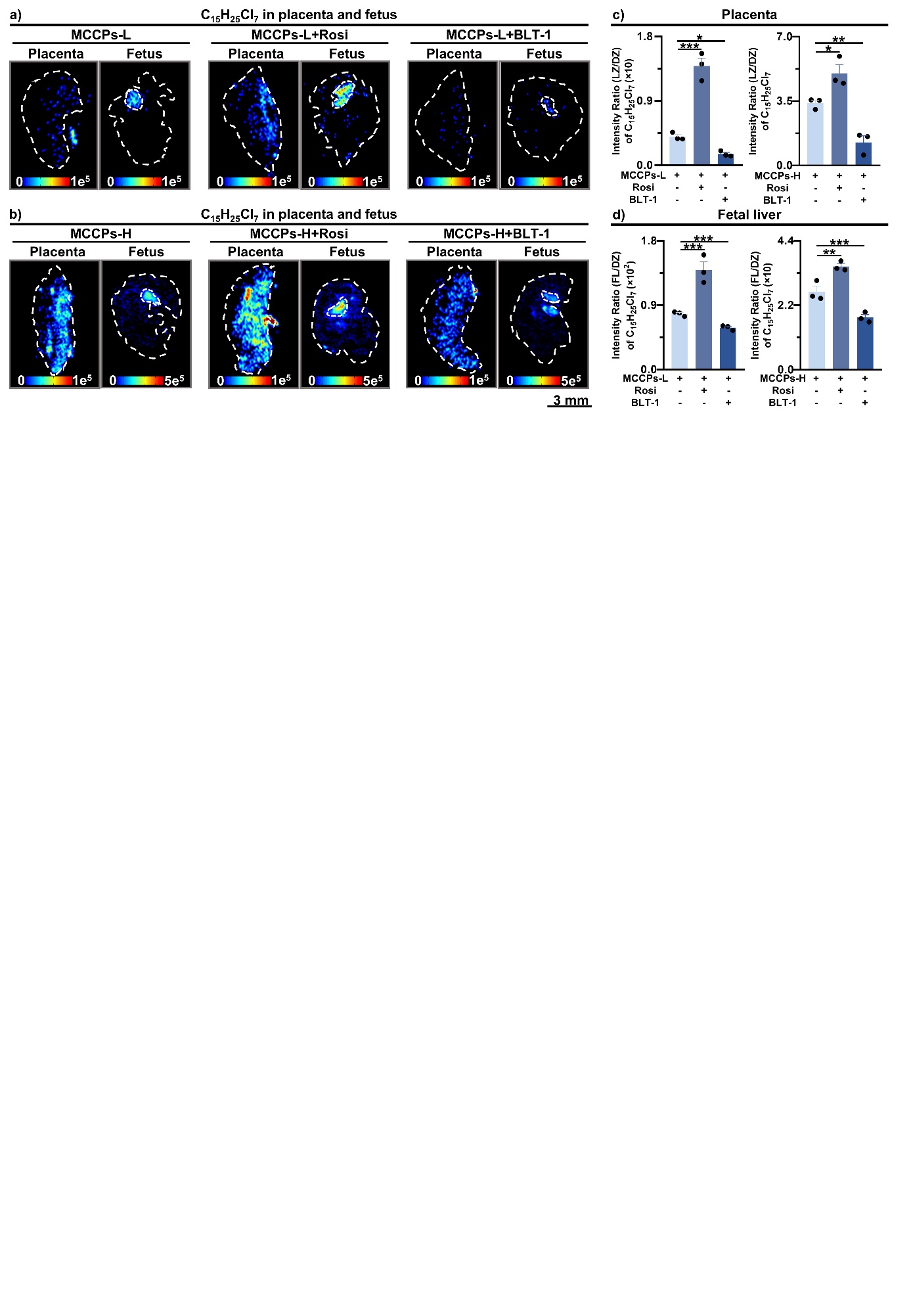


**Figure S22.** SR-B1-mediated transplacental transfer of hydrophobic MCCPs (representative congener: C_15_H_25_Cl_7_) *in vivo*. (a-b) MS images of MCCPs in the placenta and fetus at E11.5 across MCCP exposed groups. (c) Intensity ratio (LZ/DZ) of MCCPs in the placenta at E11.5 across MCCP exposed groups (*n* = 3 biological replicates per group). (d) Intensity ratio (FL/DZ) of MCCPs in FL at E11.5 across treatment groups (*n* = 3 biological replicates per group). Data represent mean ± SEM. **p* < 0.05, ***p* < 0.01, ****p* < 0.001. MCCPs, medium-chain chlorinated paraffins; Rosi, rosiglitazone; BLT-1, block lipid transport-1; DZ, decidual zone; LZ, labyrinth zone; FL, fetal liver.


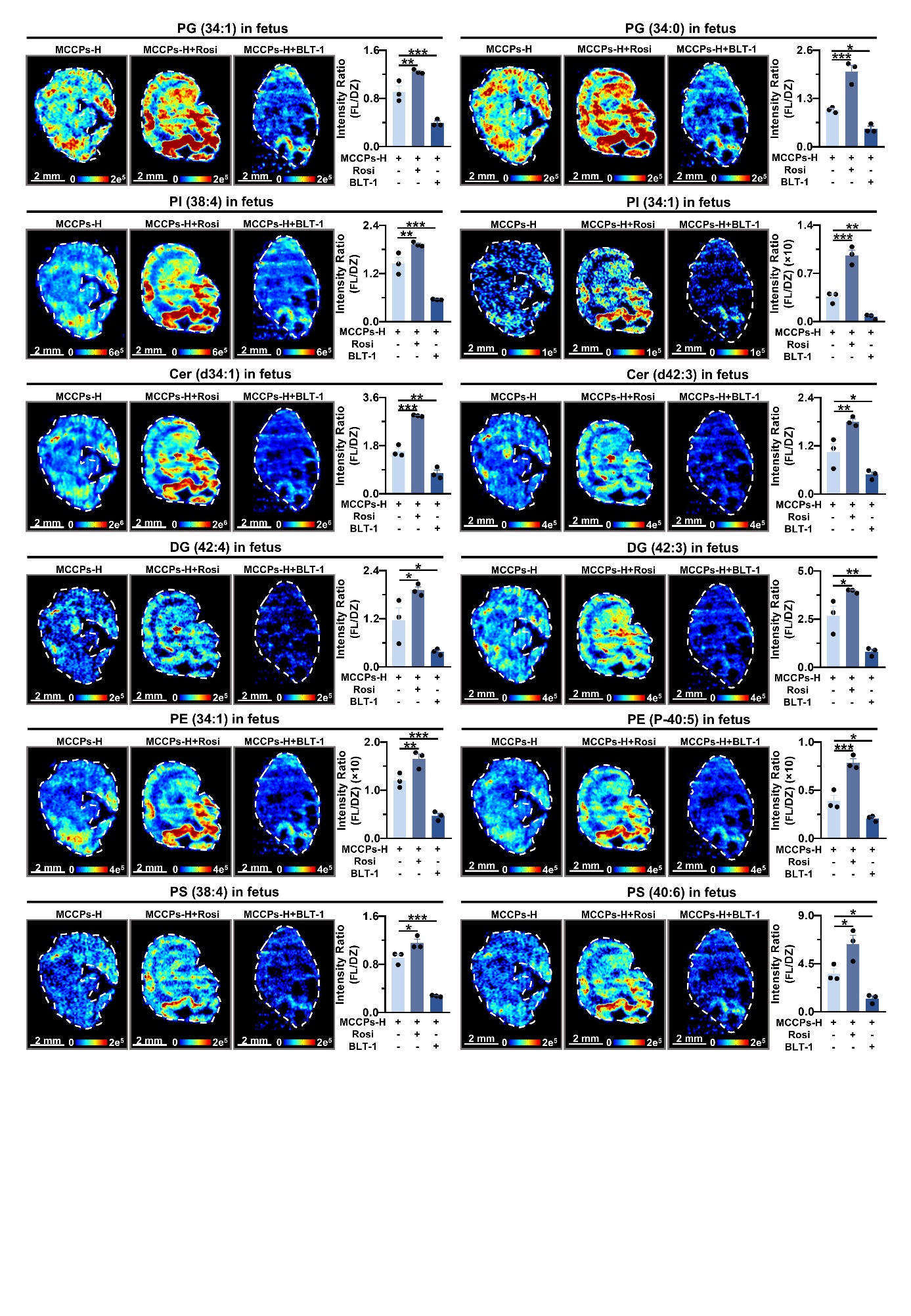


**Figure S23.** MS images and intensity ratio (FL/DZ) of lipids in fetus at E11.5 across MCCP high-dose treatment groups (*n* = 3 biological replicates per group). Data represent mean ± SEM. **p* < 0.05, ***p* < 0.01, ****p* < 0.001. MCCPs, medium-chain chlorinated paraffins; Rosi, rosiglitazone; BLT-1, block lipid transport-1; PG, phosphatidylglycerol; PI, phosphatidylinositol; Cer, ceramide; DG, diacylglycerol; PE, phosphatidylethanolamine; PS, phosphatidylserine; FL, fetal liver.


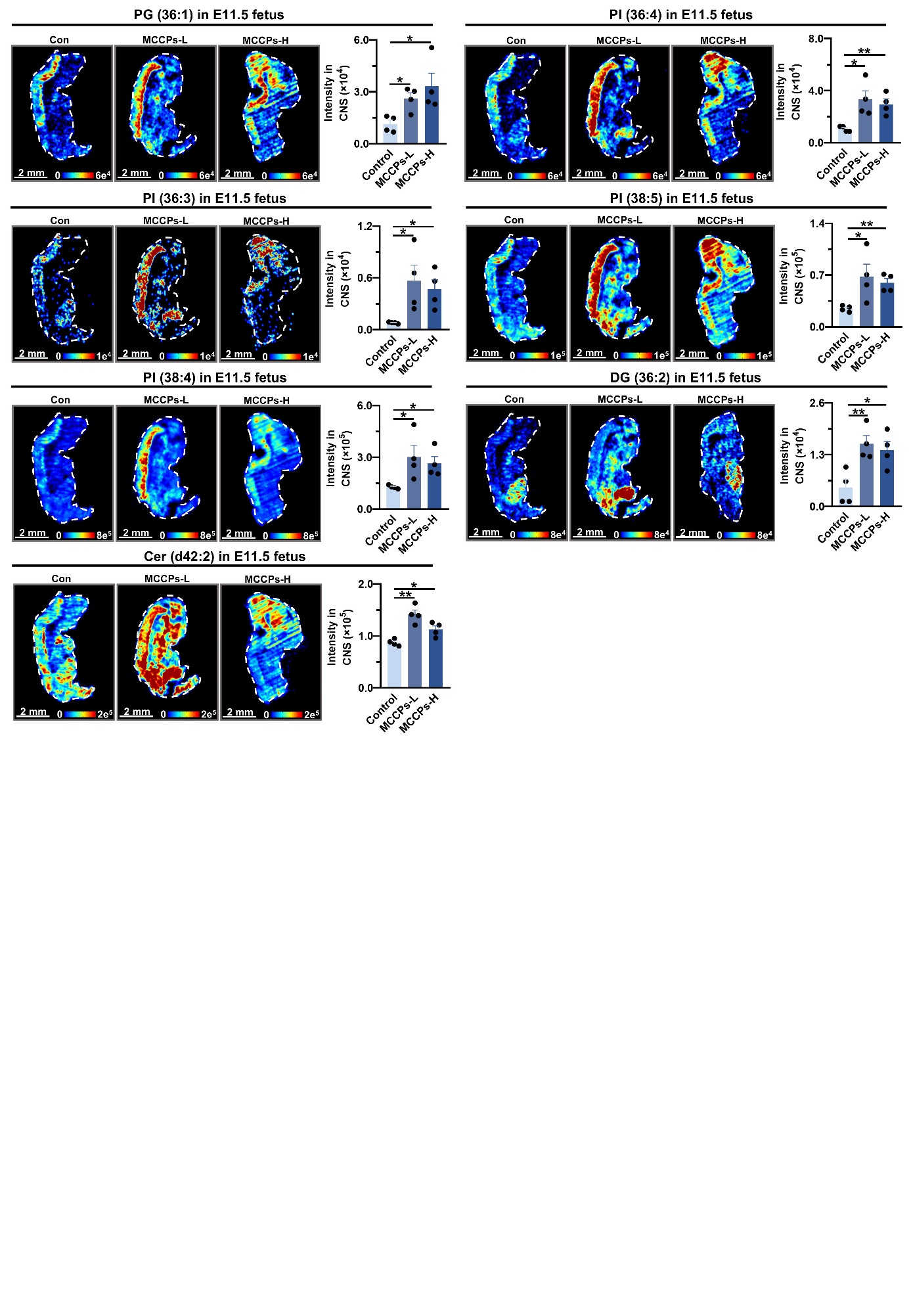


**Figure S24.** MS images and intensity of toxic lipids in CNS of fetus at E11.5 across MCCP exposed groups (*n* = 4 biological replicates per group). Data represent mean ± SEM. **p* < 0.05, ***p* < 0.01, ****p* < 0.001. MCCPs, medium-chain chlorinated paraffins; PG, phosphatidylglycerol; PI, phosphatidylinositol; DG, diacylglycerol; Cer, ceramide; CNS, central nervous system.


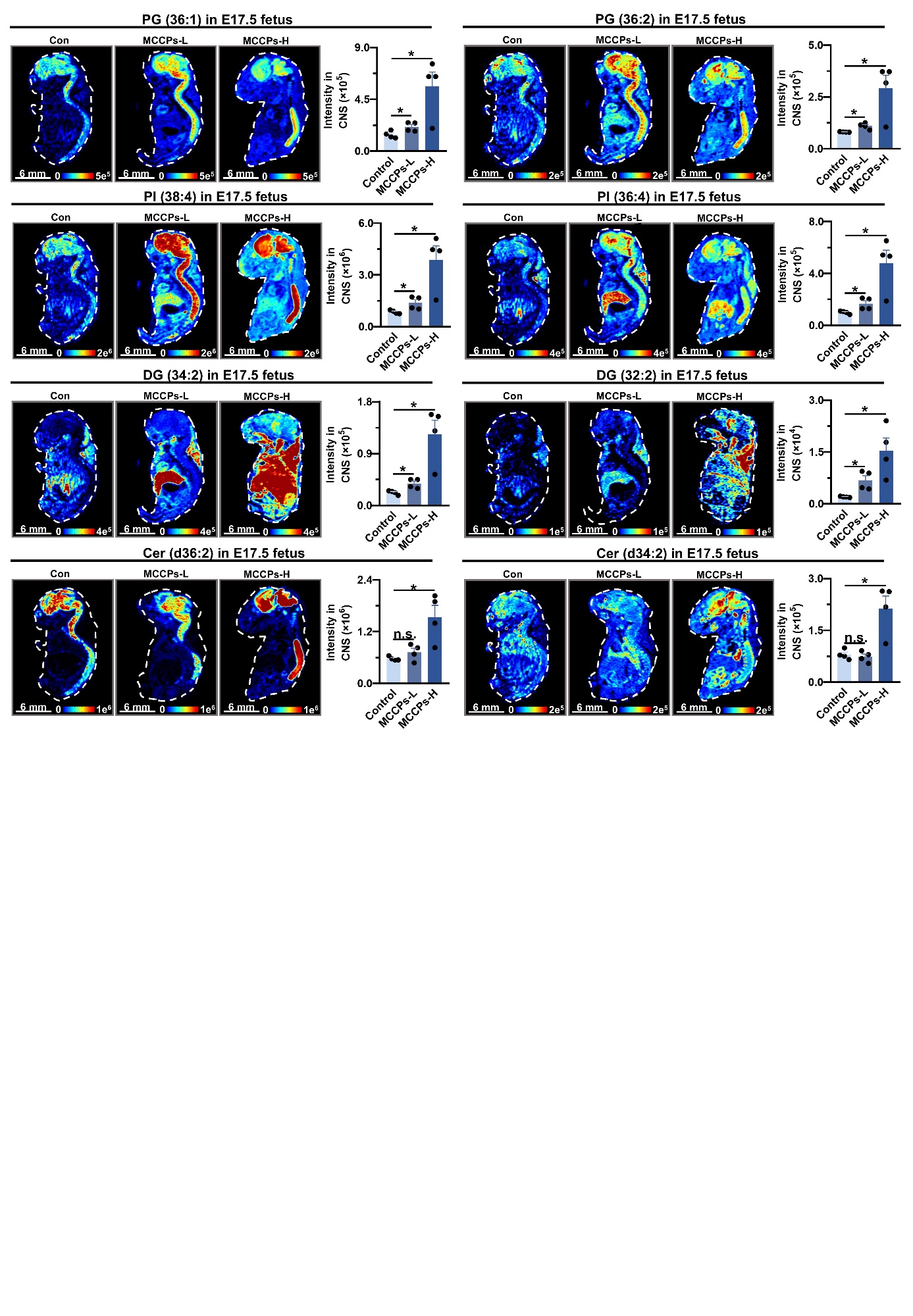


**Figure S25.** MS images and intensity of toxic lipids in CNS of fetus at E17.5 across MCCP exposed groups (*n* = 4 biological replicates per group). Data represent mean ± SEM. n.s., not significant; **p* < 0.05, ***p* < 0.01, ****p* < 0.001. MCCPs, medium-chain chlorinated paraffins; PG, phosphatidylglycerol; PI, phosphatidylinositol; DG, diacylglycerol; Cer, ceramide; CNS, central nervous system.


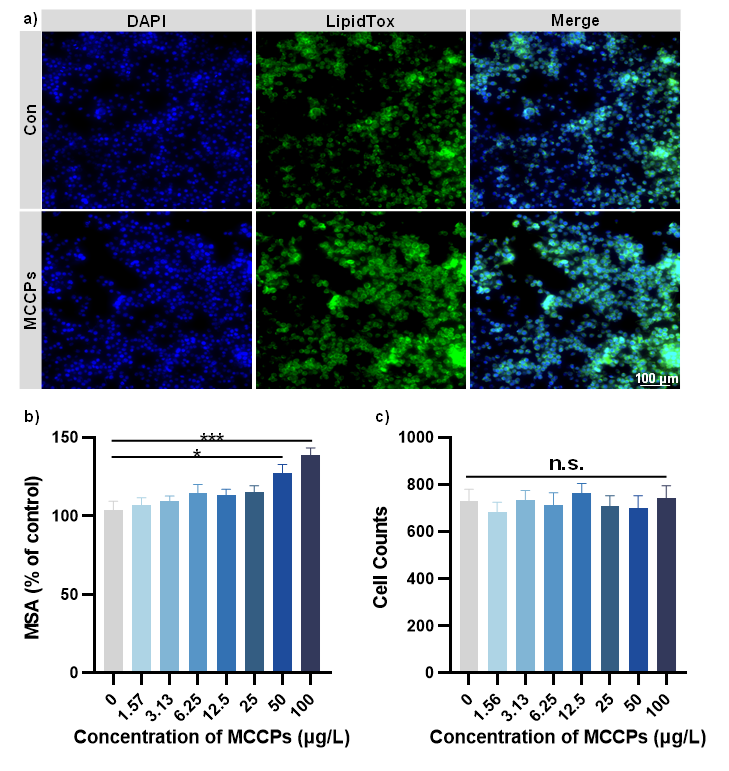


**Figure S26.** MCCPs-induced lipotoxicity in BV2 cells. (a-b) Representative images (a) and levels (b) of HCS LipidTOX^TM^ Green in BV2 cells treated with MCCPs (*n* = 3 biological replicates per group). (c) Cell counts across treatment groups (*n* = 3 biological replicates per group). Data represent mean ± SEM. n.s., not significant; **p* < 0.05, ***p* < 0.01, ****p* < 0.001. MCCPs, medium-chain chlorinated paraffins; MSA, mean stained area.


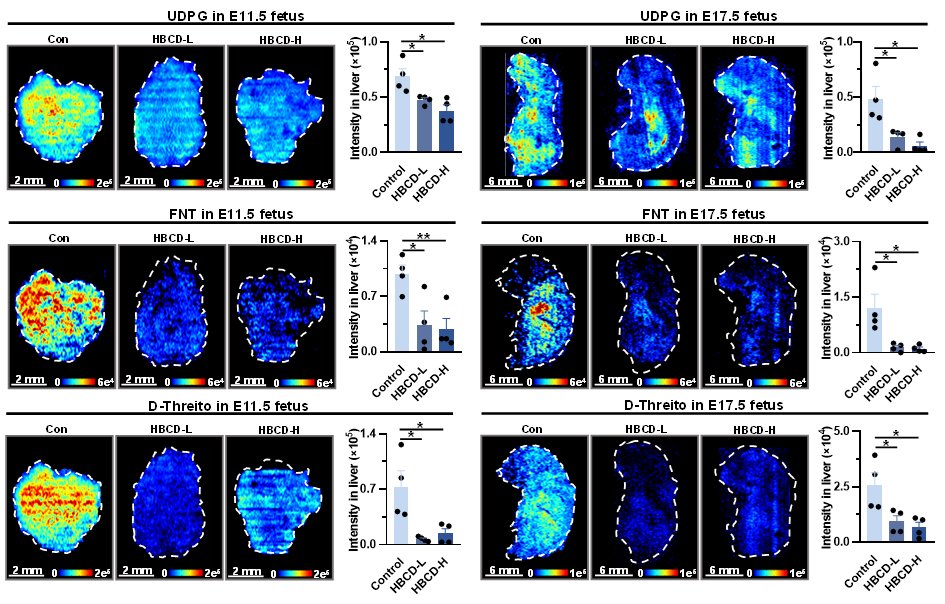


**Figure S27.** MS images and intensity of differential metabolites in fetus at E11.5 and E17.5 across HBCD exposed groups (*n* = 4 biological replicates per group). Data represent mean ± SEM. **p* < 0.05, ***p* < 0.01, ****p* < 0.001. HBCD, hexabromocyclododecane; UDPG, uridine diphosphate glucose; FNT, formamidopyrimidine nucleoside triphosphate.
